# Novel lncRNA regulatory elements in milk somatic cells of Holstein dairy cows associated with mastitis

**DOI:** 10.1038/s42003-024-05764-y

**Published:** 2024-01-15

**Authors:** Victoria Asselstine, Juan F. Medrano, Malane M. M. Muniz, Bonnie A. Mallard, Niel A. Karrow, Angela Cánovas

**Affiliations:** 1 Centre for Genetic Improvement of Livestock, Department of Animal Biosciences, University of Guelph, N1G 2W1 Guelph, ON Canada; 2 Department of Animal Science, University of California-Davis, 95616 Davis, CA USA; 3 Department of Pathobiology, Ontario Veterinary College, University of Guelph, N1G 2W1 Guelph, ON Canada

**Keywords:** Functional genomics, Gene regulation

## Abstract

Despite regulatory elements such as long non - coding RNAs representing most of the transcriptome, the functional understanding of long non - coding RNAs in relation to major health conditions including bovine mastitis is limited. This study examined the milk somatic cell transcriptome from udder quarters of 6 Holstein dairy cows to identify differentially expressed long non - coding RNAs using RNA - Sequencing. Ninety - four differentially expressed long non - coding RNAs are identified, 5 of which are previously annotated for gene name and length, 11 are annotated for gene name and 78 are novel, having no gene name or length previously annotated. Significant inflammatory response and regulation of immune response pathways (false discovery rate < 0.05) are associated with the differentially expressed long non - coding RNAs. QTL annotation analysis revealed 31 QTL previously annotated in the genomic regions of the 94 differentially expressed long non - coding RNAs, and the majority are associated with milk traits. This research provides a better understanding of long non - coding RNAs regulatory elements in milk somatic cells, which may enhance current breeding strategies for more adaptable or high mastitis resistant cattle.

## Abstract (web summary)

RNA-seq analysis of lncRNAs in Holstein dairy cows identifies lncRNA functional regulatory elements in milk somatic cells, which in turn may enhance cattle breeding strategies for mastitis resistance.

## Introduction

Bovine mastitis is a highly prevalent disease of lactating dairy cows that results in milk yield reduction, discarded milk, and early culling. Mastitis can be classified as clinical or subclinical, with clinical status defined as changes in the milk, such as flakes and clotting or inflammation in the udder^1^; and subclinical, status defined as an elevated level of milk somatic cells (SC) which consist of macrophages, polymorphonuclear leukocytes, lymphocytes, and epithelial cells^2,3^. Although abundant research exists on cattle mastitis, it remains a major economic challenge for dairy producers due to the complexity of the trait.

With the advent of RNA-sequencing (RNA-Seq), the highly dynamic host transcriptome can be studied at a high-throughput level to identify key molecular differences between healthy and mastitic animals, such as genes, transcripts, and regulatory elements. The host transcriptome consists of both protein-coding and non-coding genes. Messenger RNAs are protein-coding RNAs and well-studied; however, they account for <2% of the total genomic sequence, illustrating that most transcripts are non-coding^4^. More specifically, non-coding transcripts such as long non-coding RNAs (lncRNAs), defined as transcripts longer than 200 nucleotides in length with limited to no coding capacity^5^, implement diverse cellular and biological functions through a multiplicity of biochemical activities, including transcriptional and post-transcriptional processing^6^. The lncRNA is classified as *cis*-acting, where they recognize complementary sequences of genes in close proximity (i.e., neighboring genes) and carry out their functions there, or *trans*-acting, where the lncRNA is transcribed, processed, and then vacate their sites of transcription to exert their functions elsewhere^7^. Regardless of functional region, it is crucial that lncRNA recognize complementary sequences of their targets to allow specific interactions to occur, such as editing, translation, degradation, splicing, and transport^8^.

Previous research using RNA-Seq aimed to identify and annotate novel lncRNAs in the transcriptome of 18 bovine tissues from a single animal, including lung, liver, kidney, white blood cells, and mammary gland^9^. The latter study revealed that kidney, liver, and lung tissue have large proportion of unknown transcripts (53%, 52%, and 48%, respectively), whereas the mammary gland has only 13.13% unknown transcripts. However, lncRNA expression is highly tissue and time-specific, and many are only detected under stress conditions^10^. Studying the lncRNA in the bovine mammary gland under stress conditions has revealed their involvement in many biological functions including susceptibility to clinical mastitis^5^. Wang et al.^11^ studied novel lncRNA expressed in bovine tissues and found that the novel *lncRNA-TUB* was expressed at higher concentrations in mammary epithelial cells that received a pro-inflammatory stimulus in comparison to normal cells. Therefore, if the lncRNAs have a fundamental role in immune regulation due to their interactions with transcripts in close proximity, this may provide key insights regarding mastitis resistance, which will aid in the development of breeding programs for mastitis.

The objectives of this study were to: (1) detect lncRNAs present in the bovine milk SC transcriptome of 6 healthy and 6 mastitic samples collected from 6 Holstein dairy cows using RNA-Seq; (2) identify lncRNAs that are differentially expressed (DE) between healthy and mastitic samples; (3) classify previously annotated lncRNAs and identify novel lncRNAs having no previously annotated gene name or length; (4) perform functional analysis to determine if these lncRNAs acting in close proximity to mRNA, miRNA etc. are associated with the host immune response or mastitis resistance; (5) perform QTL annotation and QTL enrichment analysis using the genomic regions of the DE lncRNAs to find additional positional evidence of their involvement in immune response or mastitis resistance.

This study identified 94 DE lncRNAs between healthy and mastitic samples, and these DE lncRNAs were significantly involved in functional metabolic pathways (false discovery rate (FDR) < 0.05) such as inflammatory response and regulation of immune response. It was also found that 31 QTL were annotated in the genomic regions of the 94 DE lncRNAs and the majority of these were associated with milk traits. This research provides a better understanding of lncRNA regulatory elements in the transcriptome of bovine milk SC, which may help to improve the selection of cows better able to adapt or be more resistant to mastitis.

## Results and discussion

### Differential lncRNA expression analysis and classification

A total of 182 million single-end reads were generated from the 12 milk SC samples. RNA - Sequencing analysis revealed that 90% (87% healthy; 93% mastitic) of these reads were uniquely mapped to the bovine reference genome (ARS - UCD1.2.1) using a Large Gap Read Mapping (LGRM) approach (Supplementary Table 1). As shown in Supplementary Table 1, differences in the total mapped reads between the samples was observed. This may be due to the different cell types present in mastitic vs healthy udder quarters, as isolation of different cell types is a common limitation to transcriptomic studies^12–14^. Although the bovine reference genome currently has 27,607 annotated transcripts, our analysis revealed a total of 31,870 and 29,154 transcripts in healthy and mastitic samples, respectively (reads per kilobase per million mapped reads (RPKM) ≥ 0.2). This difference accounts for the identification of novel transcripts not yet annotated. In total, 659 transcripts were DE between healthy and mastitic samples (FDR < 0.05 and fold-change ( | FC | ) > 2). After filtering these transcripts for only DE lncRNA, 94 DE lncRNAs between healthy and mastitic samples were identified. These lncRNAs were then further categorized based on previous annotation in the bovine reference genome; 5 being previously annotated for gene name and length, 11 being previously annotated for gene name, whereas the majority (*N* = 78) were novel with no annotated gene name or length.

### Annotation of long non-coding RNA

The lncRNA were categorized as previously annotated when they had both a specific Ensembl identifier (e.g., *ENSBTAG00000013417*) and the length corresponded to or was different from that reported in the ARS_UCD1.2.1 bovine reference genome. Of the 94 DE lncRNAs, only 5 of the identified DE lncRNAs were previously annotated for both gene name and length in the bovine reference genome (Table 1), whereas 11 DE lncRNAs had an associated gene name, but the length was different than that reported in the bovine reference genome (Table 2). Therefore, there were 16 DE lncRNAs classified as previously annotated; of these, 14 were over-expressed and 2 were under-expressed in the mastitic samples compared to the healthy samples. Although these 16 DE lncRNAs were previously annotated, they do not currently have a gene symbol assigned to them; therefore, all functional analysis was completed using their Ensembl identifier. The most over-expressed DE lncRNA in the mastitic samples compared to the healthy samples was *ENSBTAG00000070418_2* (FC = 52.90, FDR = 1.01E-02). This lncRNA was annotated for gene name only as the length is different than what is reported in the bovine reference genome. This lncRNA predicted target is phosphoinositide 3-kinase regulatory subunit 4 (*PIK3R4*) based on its downstream proximity in the bovine reference genome (Table 2). This interaction was significantly negatively correlated (r = −0.82, *P*-value = 0.05; Supplementary Table 2) with the specific mRNA isoform of *PIK3R4_3* in the healthy group. This gene family (PI3K) is involved in many aspects of immune cell development including regulatory T cells which are important in regulating and suppressing other cells in the immune system^15^. Therefore, if the lncRNA/gene interaction is altered, this could negatively impact the host’s immune response. Alternatively, the most under-expressed lncRNA (annotated for gene name only) in the mastitic samples was *ENSBTAG00000082333* (FC = −39.49, FDR = 4.34E-02). This lncRNA predicted target interaction is with kappa casein (*CSN3*), which is located upstream of the lncRNA in the bovine reference genome. This specific casein constitutes about 25% of the casein fraction in bovine milk^16^. It also determines the size of casein micelles and initiates micelle aggregation for cheese production^17^. Thus, if the lncRNA / gene interaction is altered, this could in turn negatively affect the *CSN3* protein that is formed. However, it was found that in our dataset, the correlation between the expression levels of these isoforms was not significant.

**Table 1:** Differentially expressed lncRNAs from the 12 milk somatic cell samples collected from 6 Holstein dairy cows, previously annotated for both gene name and length in the bovine reference genome ARS - UCD1.2.1, with their predicted interaction.

lncRNA ID	Position	FC (log10)^a^	FDR	Location^b^	Predicted interaction^c^
*ENSBTAG00000050065_2*	20:70,033,246–70,035,651	−35.43	4.80E-02	Overlapping	*IRX2*
*ENSBTAG00000054150_1*	22:53,288,521–53,302,341	13.61	4.94E-03	Overlapping	*XCR1*
*ENSBTAG00000051221_3*	14:14,617,953–14,648,986	16.08	4.66E-02	Downstream	*TRIB1*
*ENSBTAG00000052589_1*	1:151,342,531–151,427,134	16.31	4.53E-02	Downstream	*PIK3R4*
*ENSBTAG00000050727_2*	20:28,801,242–28,823,133	19.02	3.28E-02	Upstream	*bta-mir-10163*
				Upstream	*EMB*

^a^FC (log10) = Fold change (log10).

^b^Location is defined as the localization of the predicted interaction as shown in Genome Data Viewer (https://www.ncbi.nlm.nih.gov/genome/gdv/).

^c^Predicted interaction is determined based on the proximity to the lncRNA.

**Table 2:** Differentially expressed lncRNAs from the 12 milk somatic cell samples collected from 6 Holstein dairy cows, previously annotated for gene name in the bovine reference genome ARS - UCD1.2.1, with their predicted interaction.

lncRNA ID	Position	Length	FC (log10)^a^	FDR	Location^b^	Predicted interaction^c^
*ENSBTAT00000082333*	6:85,666,003–85,671,517	3312	−39.49	1.93E-02	Overlapping	*PRPF4B*
*ENSBTAT00000072182*	15:82,272,102–82,282,761	9598	5.93	1.77E-02	Upstream	*FAM111B*
					Downstream	*DTX4*
*ENSBTAT00000084749*	13:54,696,287–54,705,035	4605	6.60	1.43E-02	Upstream	*SLCO4A1*
					Downstream	*bta-mir-133a-1*
*ENSBTAT00000086278*	X:130,716,958–130,722,792	5665	8.91	3.27E-02	Overlapping	*TLR8*
*ENSBTAT00000068605*	14:14,620,109–14,626,476	4863	19.82	2.37E-02	Downstream	*TRIB1*
*ENSBTAT00000069074*	20:28,791,624–28,829,693	18,089	25.06	4.09E-03	Upstream	*bta-mir-10163*
					Upstream	*EMB*
*ENSBTAT00000070418_1*	1:151,403,342–151,411,502	7694	25.41	3.06E-03	Downstream	*PIK3R4*
*ENSBTAT00000070418_4*	1:151,346,789–151,359,836	10,958	27.38	3.14E-03	Downstream	*PIK3R4*
*ENSBTAT00000084307*	9:72,480,486–72,491,143	4285	45.79	2.16E-03	Upstream	*SGK1*
*ENSBTAT00000070418_3*	1:151,346,789–151,359,836	10,985	46.89	4.37E-02	Downstream	*PIK3R4*
*ENSBTAT00000070418_2*	1:151,419,045–151,427,963	7964	52.90	1.01E-02	Downstream	*PIK3R4*

^a^FC (log10) = Fold change (log10).

^b^Location is defined as the localization of the predicted interaction as shown in Genome Data Viewer (https://www.ncbi.nlm.nih.gov/genome/gdv/).

^c^Predicted interaction is determined based on the proximity to the lncRNA.

### Identification of novel long non-coding RNA

Approximately 83% of the DE lncRNAs (78/94 lncRNAs) identified in this study were classified as novel as they were not previously annotated for gene name or length in the bovine reference genome (Table 3). We identified the potential transcript interaction of these novel lncRNAs relative to their position to other transcripts in the genome. For example, transcripts that were close to an annotated mRNA or overlapped with an annotated mRNA were inferred as the target of the lncRNA in relation to its functionality within the genome. As shown in Table 3, most of the novel DE lncRNAs were found to be intergenic (71.79%), whereas 28.21% were genic. Previous research has demonstrated that bovine lncRNA are mostly located in intergenic regions; however, this is commonly found in muscle tissues^9,18,19^.

**Table 3:** Differentially expressed novel lncRNAs from the 12 milk somatic cell samples collected from 6 Holstein dairy cows and their predicted interaction.

Type^a^	lncRNA ID	Position	FC (log10)^b^	FDR	Subtype^c^	Location^d^	Predicted interaction^e^
Genic	*lncRNA_68.2*	2:69,536,597–69,537,404	−170.31	4.85E-02	nested	Exonic	*ENSBTAG00000016785*
	*lncRNA_2142.1*	X:18,824,232–18,824,648	−30.59	6.20E-03	nested	Intronic	*ENSBTAG00000039890*
	*lncRNA_2526.1*	8:7,566,228–7,572,723	4.87	3.69E-02	nested	Intronic	*CTSB*
	*lncRNA_577.4*	10:73,875,845–73,886,496	5.59	4.47E-02	overlapping	Exonic	*HIF1A*
	*lncRNA_577.1*	10:73,857,290–73,886,496	6.93	4.55E-02	overlapping	Exonic	*HIF1A*
	*lncRNA_2570.1*	9:72,403,793–72,417,951	7.01	4.87E-02	nested	Intronic	*SGK1*
	*lncRNA_2901.1*	16:75,129,679–75,141,399	7.12	4.81E-02	nested	Intronic	*PLXNA2*
	*lncRNA_1930.1*	24:2,244,864–2,267,849	7.87	3.70E-02	nested	Intronic	*MBP*
	*lncRNA_445.5*	8:39,117,170–39,121,520	9.24	4.15E-02	nested	Intronic	*PDCD1LG2*
	*lncRNA_24.1*	1:79,582,639–79,587,015	9.50	2.44E-02	nested	Intronic	*BCL6*
	*lncRNA_2188.5*	1:79,578,299–79,586,675	10.28	4.72E-02	nested	Intronic	*BCL6*
	*lncRNA_3260.1*	25:10,268,031–10,272,270	10.63	2.47E-02	nested	Intronic	*LITAF*
	*lncRNA_2312.1*	3:86,264,056–86,271,860	10.82	4.28E-02	nested	Intronic	*FGGY*
	*lncRNA_2824.1*	14:8,219,532–8,233,156	10.96	1.38E-02	nested	Intronic	*TG*
	*lncRNA_2188.3*	1:79,578,299–79,586,675	12.01	3.43E-02	nested	Intronic	*BCL6*
	*lncRNA_2518.1*	7:72,212,755–72,237,929	13.00	3.22E-03	overlapping	Intronic	*ATP10B*
	*lncRNA_3370.1*	29:46,222,797–46,225,081	13.65	4.02E-03	nested	Intronic	*CPT1A*
	*lncRNA_3349.1*	29:39,786,343–39,787,254	20.48	3.60E-02	nested	Intronic	*VWCE*
	*lncRNA_2311.3*	3:86,227,511–86,228,613	20.83	4.52E-02	nested	Intronic	*FGGY*
	*lncRNA_1751.3*	19:48,405,247–48,407,474	26.13	4.52E-02	nested	Intronic	*TEX2*
	*lncRNA_2311.2*	3:86,227,432–86,235,257	62.74	4.94E-03	antisense	Intronic	*FGGY*
	*lncRNA_1505.1*	16:44,572,821–44,616,632	496.05	1.20E-02	containing	Intronic	*U6*
Intergenic	*lincRNA_64.1*	2:31,591,445–31,598,613	−41.23	3.69E-03	same_strand	Downstream	*COBLL1*
	*lincRNA_462.2*	8:66,935,638–66,944,722	−12.11	3.22E-02	divergent	Upstream	*LPL*
	*lincRNA_1668.5*	18:62,448,169–62,451,204	4.55	2.25E-02	same_strand	Downstream	*ENSBTAG00000046383*
	*lincRNA_1670.2*	18:62,480,278–62,482,466	4.94	4.35E-02	same_strand	Upstream	*ENSBTAG00000045854*
	*lincRNA_2814.2*	13:78,085,194–78,090,510	6.24	4.85E-02	same_strand	Downstream	*UBE2V1*
	*lincRNA_3134.2*	21:25,998,499–26,007,306	6.27	3.56E-02	divergent	Upstream	*BCL2A1*
	*lincRNA_1664.4*	18:61,084,423–61,092,475	6.32	4.87E-02	same_strand	Upstream	*ENSBTAG00000014953*
	*lincRNA_1668.4*	18:62,445,176–62,453,609	6.42	1.17E-02	same_strand	Downstream	*ENSBTAG00000046383*
	*lincRNA_3309.2*	26:43,832,040–43,845,425	6.71	4.34E-02	divergent	Upstream	*ENSBTAG00000049849*
	*lincRNA_1450.2*	15:6,406,173–6,411,563	6.88	1.60E-02	same_strand	Upstream	*BIRC3*
	*lincRNA_2174.2*	1:2,129,653–2,161,384	7.12	4.19E-02	divergent	Upstream	*IFNGR2*
	*lincRNA_2592.2*	10:17,316,443–17,343,718	7.19	4.53E-02	same_strand	Downstream	*bta-mir-2285ar*
	*lincRNA_289.5*	5:101,489,332–101,503,518	7.20	4.24E-02	divergent	Upstream	*SLC2A3*
	*lincRNA_575.1*	10:70,577,812–70,592,366	7.31	1.99E-02	divergent	Upstream	*ARID4A*
	*lincRNA_2868.1*	15:77,662,951–77,671,785	7.39	3.11E-02	same_strand	Upstream	*PTPRJ*
	*lincRNA_2254.2*	2:128,096,244–128,103,690	8.09	4.43E-02	convergent	Downstream	*CLIC4*
	*lincRNA_1523.4*	16:73,637,687–73,641,729	8.30	1.01E-02	same_strand	Upstream	*HSD11B1*
	*lincRNA_2816.2*	13:78,397,999–78,417,535	8.49	2.13E-02	same_strand	Downstream	*ENSBTAG00000048499*
	*lincRNA_200.6*	3:119,431,372–119,434,064	8.62	4.31E-02	same_strand	Downstream	*CSF2RA*
	*lincRNA_2290.2*	3:19,176,164–19,181,274	8.64	3.57E-02	same_strand	Upstream	*ENSBTAG00000054946*
	*lincRNA_495.7*	9:72,491,395–72,495,171	8.80	3.11E-02	same_strand	Downstream	*ENSBTAG00000048577*
	*lincRNA_2900.2*	16:73,644,521–73,661,286	9.15	1.20E-02	same_strand	Upstream	*LAMB3*
	*lincRNA_2586.2*	10:4,275,621–4,325,006	9.19	4.47E-02	convergent	Downstream	*ENSBTAG00000054905*
	*lincRNA_1874.4*	22:53,009,866–53,015,577	9.64	1.89E-02	same_strand	Upstream	*CCRL2*
	*lincRNA_2178.2*	1:6,978,767–6,991,886	10.06	2.37E-02	convergent	Downstream	*MAP3K7CL*
	*lincRNA_3099.2*	19:61,054,956–61,062,798	10.20	1.02E-02	same_strand	Upstream	*ENSBTAG00000049077*
	*lincRNA_3204.1*	23:15,178,687–15,184,156	10.25	1.59E-02	same_strand	Upstream	*TREM1*
	*lincRNA_2165.1*	X:98,097,707–98,098,234	10.29	3.43E-02	same_strand	Upstream	*bta-mir-222*
	*lincRNA_2394.2*	5:68,002,142–68,012,805	10.31	1.08E-02	same_strand	Upstream	*CHST11*
	*lincRNA_2911.1*	17:40,237,014–40,239,978	10.58	4.83E-02	divergent	Upstream	*FNIP2*
	*lincRNA_2828.2*	14:14,705,346–14,709,348	10.62	1.96E-02	same_strand	Upstream	*ENSBTAG00000051221*
	*lincRNA_3182.1*	22:53,273,501–53,288,313	10.66	4.47E-02	divergent	Upstream	*ENSBTAG00000054150*
	*lincRNA_3106.2*	19:61,983,057–61,988,443	10.94	1.92E-03	divergent	Upstream	*ENSBTAG00000054225*
	*lincRNA_3181.2*	22:53,243,326–53,273,444	11.07	7.52E-03	same_strand	Downstream	*ENSBTAG00000052050*
	*lincRNA_46.2*	1:151,435,865–151,447,158	11.12	3.05E-02	same_strand	Upstream	*ENSBTAG00000052589*
	*lincRNA_2411.6*	5:104,022,584–104,023,733	11.31	3.19E-02	divergent	Upstream	*TNFRSF1A*
	*lincRNA_575.2*	10:70,577,812–70,592,366	12.28	4.08E-02	divergent	Upstream	*ARID4A*
	*lincRNA_2517.4*	7:72,116,946–72,140,964	13.35	4.67E-02	same_strand	Downstream	*bta-mir-146a*
	*lincRNA_2580.2*	9:95,972,050–95,974,378	13.57	1.06E-02	same_strand	Upstream	*SOD2*
	*lincRNA_2289.2*	3:19,175,438–19,178,492	13.83	3.49E-02	divergent	Upstream	*ENSBTAG00000054946*
	*lincRNA_2788.1*	13:41,929,557–41,932,784	15.06	2.10E-02	same_strand	Downstream	*ENSBTAG00000049546*
	*lincRNA_2517.2*	7:72,110,607–72,140,964	15.49	2.54E-03	same_strand	Downstream	*bta-mir-146a*
	*lincRNA_3224.2*	23:28,259,076–28,262,553	16.09	5.41E-03	divergent	Upstream	*IER3*
	*lincRNA_3181.3*	22:53,243,340–53,273,444	16.81	1.90E-02	same_strand	Downstream	*ENSBTAG00000052050*
	*lincRNA_3324.2*	28:8,656,829–8,661,856	17.14	1.47E-02	same_strand	Downstream	*NID1*
	*lincRNA_46.6*	1:151,438,938–151,442,397	17.70	1.37E-03	same_strand	Upstream	*ENSBTAG00000052589*
	*lincRNA_2310.2*	3:86,164,085–86,182,808	18.26	4.50E-04	same_strand	Upstream	*HOOK1*
	*lincRNA_2780.2*	12:86,575,607–86,578,827	18.40	9.85E-03	convergent	Downstream	*CUL4A*
	*lincRNA_46.4*	1:151,435,865–151,447,158	26.58	1.43E-02	same_strand	Upstream	*ENSBTAG00000052589*
	*lincRNA_2322.5*	3:104,443,741–104,466,628	34.54	3.34E-02	divergent	Upstream	*ENSBTAG00000053979*
	*lincRNA_2963.2*	18:45,896,930–45,899,535	41.88	4.41E-02	same_strand	Downstream	*FXYD5*
	*lincRNA_343.3*	6:115,952,167–115,960,221	44.62	1.55E-03	divergent	Upstream	*SH3BP2*
	*lincRNA_498.1*	9:87,117,748–87,120,256	168.93	1.50E-02	same_strand	Downstream	*ENSBTAG00000038891*
	*lincRNA_2140.1*	X:4,467,170–4,473,739	197.51	2.02E-02	convergent	Downstream	*ENSBTAG00000006252*
	*lincRNA_3210.4*	23:17,592,282–17,611,254	291.66	4.66E-02	convergent	Downstream	*ENSBTAG00000050249*
	*lincRNA_2159.2*	X:79,073,608–79,080,933	683.48	4.57E-02	convergent	Downstream	*CXCR3*

^a^lncRNA type defined according to the overlap with nearest transcripts from the reference annotation.

^b^FC (log10) = Fold change (log10).

^c^subtype defined according to the orientation of the interactions.

^d^location defined as the localization of the interactions.

^e^preditcted interaction is the transcript that the lncRNA is closest to in the bovine reference annotation (ARS.UCD1.2.100), transcripts can be mRNA, miRNA, lncRNA, snRNA, or pseudogene.

### Intergenic non-coding RNA

As mentioned, 71.79% of the novel DE lncRNAs identified in this study were long intergenic non-coding RNA; (lincRNA; N = 56), meaning they are located upstream or downstream between genes^20^. The most under-expressed novel DE lincRNA was *lincRNA_64.1* (FC = − 41.23, FDR = 3.69E-03). Based on its downstream position in the bovine genome, it is predicted to interact with cordon - bleu WH2 repeat protein like 1 (*COBLL1*). The expression levels between *linc_64.1* and *COBLLI* are significantly negatively correlated (*r* = −0.86, *P*-value = 0.03) for the specific mRNA isoform *COBLLI_3* in the healthy group (Supplementary Table 2). The *COBLL1* gene has been linked with metritis, which is an inflammatory condition of the uterus, generally caused by a bacterial infection after calving when the cows have a suppressed immune system and are more vulnerable to bacterial infection^21,22^. This is interesting to consider, as some post-calving diseases also indicate an animal’s susceptibility to mastitis. Research by Thompson-Crispi et al.^23^ demonstrated high immune responding cows had less mastitis, along with metritis, retained placenta, and displaced abomasum^23^. Additionally, a positive genetic correlation (0.19–0.49) between mastitis and other diseases such as milk fever, ketosis, and retained placenta exist^24^. Therefore, if the focus is breeding on immune responsiveness, this could decrease the overall disease occurrence^25^. As the lincRNA that potentially interacts with this *COBLL1* gene was under-expressed, this could impact the functionality of the mRNA, making the animal more susceptible to metritis and in turn mastitis. Alternatively, the most over-expressed (FC = 683.48, FDR = 4.57E-02) novel lncRNA in the mastitic samples compared to the healthy samples was *lincRNA_2159.2*. This novel lncRNA in cattle was predicted to interact with C - X - C motif chemokine receptor 3 (*CXCR3*), which is located downstream from the lncRNA. The correlation between expression values was not significant in our dataset. Chemokines are a large family of cytokines and their receptors play an important role in the recruitment, activation, and differentiation of immune cells^26^. Previous research identified 3 polymorphisms associated with subclinical mastitis within the chemokine receptor C - X - C motif chemokine receptor 2 (*CXCR2*)^27^. Based on the current literature, no polymorphisms associated with mastitis have been found in CXCR3. Therefore, further research is needed to determine if polymorphisms in this gene might impact the lncRNA / gene relationship and how this could potentially impact mastitis resistance.

### Genic long non-coding RNA

Alternatively, 28.21% of the novel DE lncRNAs were genic (*N* = 22), meaning they are found overlapping a gene. The most under-expressed genic lncRNA (FC = −170.31, FDR = 4.85E-02) in the mastitic samples compared to the healthy samples is the novel *lncRNA_68.2*. The *lncRNA_68.2* is predicted to interact with *ENSBTAG00000016785* as the lncRNA overlaps an exonic region. This gene does not currently have a gene symbol assigned to it for bovine; however, in mice it is the gene 5 - hydroxytryptamine receptor 5B (*Htr5b*) gene. This gene is conserved across chimpanzee, cow, and rat; however, through evolution, the conservation has been lost in humans^28^. The *Htr5b* gene product acts as a receptor for serotonin which is a neurotransmitter with vital roles in neural activities^29^. In bovine, serotonin is a potent regulator of calcium homeostasis, energy homeostasis and energy balance during lactation^30,31^. This energy balance is critical to ensure that the cow can meet the demand for milk production. Research has shown that the highest rates of mastitis occur during early lactation when most cows experience a negative energy balance^32–34^. As this lncRNA was found to overlap an exonic region of *Htr5b*, it is hypothesized that it could play a key role in regulation of this receptor. Since *lncRNA_68.2* was under-expressed in the mastitic samples, this could be a key lncRNA to target for future analysis to minimize the impact of the negative energy balance. Alternatively, the most over-expressed genic lncRNA in the mastitic samples compared to the healthy samples, was *lncRNA_1505.1* (FC = 496.05, FDR = 1.20E-02) and it was predicted to interact with U6 spliceosomal RNA (*U6*) due to it overlapping an intronic region of the gene. This transcript is the most highly conserved of the five spliceosomal RNAs and plays a catalytic role in the spliceosome and undergoes extensive structural rearrangements^35,36^. At the present time, the potential role of *U6* on bovine mastitis is unknown. Therefore, further research is needed to better understand the functionality of *lncRNA_1505.1* and its potential interaction with *U6* in relation to mastitis or the host immune response.

### Functional analysis of differentially expressed lncRNAs

Functional analysis was performed to deeply investigate the functional regulatory elements and their impact on the host’s response to mastitis causing agents. If the lncRNA is suppressing or enhancing the ability of its target (e.g., mRNA, miRNA), this could negatively impact the cow’s immune response, making her more susceptible to mastitis and prevent her from efficiently eliminating the threat. As the DE lncRNAs were split into 1) previously annotated; 2) novel intergenic and: 3) novel genic, the functional analysis follows this structure.

### Functional analysis of previously annotated lncRNAs for both gene name and length

As mentioned previously, 5 of the annotated lncRNA were annotated previously for both gene name and length (Table 1). To determine their predicted target(s), the genomic coordinates of the lncRNA and Genome Data Viewer was used (https://www.ncbi.nlm.nih.gov/genome/gdv/). Based on the proximal location of other mRNA, miRNA etc. the predicted interaction of each of these lncRNA was identified (Table 1).

The first annotated lncRNA *ENSBTAG00000050065_2* was 35 × under-expressed in the mastitic samples compared to the healthy samples (FC = −35.43, FDR = 4.80E-02; Table 1). The Iroquois homeobox 2 (*IRX2*) gene is located downstream from out DE lncRNA and therefore this is its predicted target. This family of genes has been reported to affect tumor growth, invasion and metastasis and have been closely linked to tumor progression and prognosis^37^. The potential effect of this lncRNA / gene interaction would have on mastitis, or the immune system is unknown, thus further research is needed. As shown in Supplementary Table 2, there were 2 potential mRNA isoforms of *IRX2* that demonstrated significant, positive interactions in the mastitic (*IRX2_1* (*r* = 0.93, *P*-value = 0.01); and *IRX2_2* (*r* = 0.99, *P*-value = 0.00) and healthy groups (*IRX2_2* (*r* = 0.98, *P*-value = 0.00).

The annotated lncRNA *ENSBTAG00000054150_1* was over-expressed in the mastitic samples compared to the healthy samples (FC = 13.61, FDR = 4.94E-02; Table 1). It is predicted to interact with X – C motif chemokine receptor 1 (*XCR1*) as the lncRNA overlaps with this gene. This gene is expressed on a subset of dendritic cells which are involved in antigen cross-presentation^38^. Antigen presentation is critical to ensure the cow can fight off the mastitis causing pathogens, such as *Staphylococcus aureus* (*S. aureus*). As this mRNA plays a key role in activating this part of the immune system, this lncRNA could help in the regulation of this mRNA.

The next annotated lncRNA *ENSBTAG00000051221_3* is predicted to interact with tribbles - 1 (*TRIB1*), based on the gene’s downstream proximity (Table 1). The correlation between these two was significantly positive (*r* = 0.90, *P*-value = 0.01; Supplementary Table 2). This gene has been linked to the regulation of anti-inflammatory macrophage polarization and inflammatory responses^39–42^. It also has important roles in controlling inflammatory cytokines, including the pro-inflammatory cytokine interleukin 8 (IL-8)^41^. Therefore, due to its critical roles in inflammatory responses, this is of key interest in terms of mastitis. The lncRNA, which is predicted to interact with this gene is over-expressed in the mastitic samples compared to the healthy samples (FC = 16.08, FDR = 4.66E-02) and, therefore, its over expression could act to suppress the functions of this key gene and therefore impact the hosts’ ability to regulate the inflammatory response, making the mastitis incidence more severe.

Next, the over-expressed (FC = 16.31, FDR = 4.53E-02) lncRNA, which is annotated for both gene name and length is *ENSBTAG00000052589*_1 (Table 1). This lncRNA is predicted to interact with the downstream gene *PIK3R4*. As mentioned earlier, this family of genes are involved in in many aspects of immune cell development^15^. Therefore, since this lncRNA is over-expressed in the mastitic samples, it could impact the genes expression and thus the cow’s ability to mount a proper immune response to the mastitis causing agent.

Lastly, *ENSBTAG00000050727*_2 is over-expressed in the mastitic samples (FC = 19.02, FDR = 3.28E-02; Table 1). This lncRNA has two potential target interactions based on their upstream proximity to the lncRNA. The first potential interaction is with *bta-mir-10163*. This miRNA has been previously found in the corpus luteum of pregnant animals and has numerous targets including retinoic acid receptor RXR - alpha (*RXRA*) which is involved in cell proliferation and apoptosis^43,44^. As lncRNA can act as miRNA sponges by binding miRNAs and preventing their interaction with their target, this could inhibit the miRNA regulatory function^45,46^. In relation to mastitis, the relationship between our DE lncRNA *ENSBTAG00000050727*_2 and the miRNA *bta-mir-10163* is unclear, but further research is necessary to investigate this. Additionally, this lncRNA has another potential interaction with the upstream gene embigin (*EMB*). Embigin is a transmembrane glycoprotein which belongs to the immunoglobulin superfamily^47^. It has been reported to be expressed in a variety of prostate and mammary cancer cell lines^48^. Currently, the potential impact of this gene on bovine mastitis is unknown, but as it has been expressed in mammary cancer cell lines, this is something that could be relevant to bovine mastitis. The correlation between the lncRNA and both of these predicted targets was not significant in our dataset.

### Functional analysis of previously annotated lncRNAs for gene name

In addition, there were 11 DE lncRNAs that were annotated for gene name, but length was different than the length reported in the ARS_UCD1.2.1 bovine reference genome (Table 2).

The first lncRNA, *ENSBTAT00000072182* is over-expressed in the mastitic samples (FC = 5.93, FDR = 1.77E-02) and has two predicted target interactions (Table 2). The first predicted target is located upstream and is family with sequence similarity 111 member B (*FAM111B*). This gene has been linked to multiple malignancies as it is an oncoprotein. The expression of *FAM111B* in breast cancer tissues is higher than observed in healthy tissues in humans^49^. Therefore, future research should confirm if this lncRNA / mRNA interaction could have a negative effect on the bovine mammary gland. This lncRNA is also predicted to interact with the downstream gene deltex E3 ubiquitin ligase 4 (*DTX4*), which has roles in the IFN-1 signaling pathway in innate immunity^50^. Innate immunity is the cows first line of defence against mastitis-causing agents. Therefore, if the over-expression of this lncRNA impacts the ability of the mRNA protein to form, this could negatively impact the cow’s initial response to mastitis.

Next, lncRNA *ENSBTAT00000084749* has two predicted targets due to their proximity to the lncRNA (Table 2). The first predicted target which is located upstream is solute carrier organic anion transporter family member 4A1 (*SLCO4A1*). This gene is highly expressed in colon cancers and promotes the cancers’ proliferation^51^. The second predicted target is the miRNA *bta-mir-133a-1* and is located downstream. This miRNA has been linked to human cardiac remodeling^52^, as well as in muscle development in beef cattle^53^. As shown in Table 2, this lncRNA is over-expressed in the mastitic samples (FC = 6.60, FDR = 1.43E-02); however, the relationship between this lncRNA and its predicted targets is unknown, so further research should look at the potential impact its expression may have on *SLCO4A1* and *bta-mir-133a-1*. The correlation between expression levels of the lncRNA and both of these predicted targets was observed to be not significant in our dataset.

The lncRNA *ENSBTAT00000086278* overlaps with toll-like receptor 8 (*TLR8*) and therefore this is its predicted target. No significant correlation between the two was found. The family of toll-like receptors (TLRs) are a family of highly conserved pattern-recognition receptors which are essential for host immune response^54^ and triggering the onset of the inflammatory cascade^55^. The lncRNA is over-expressed in the mastitic samples (FC = 8.91, FDR = 3.27E-02), therefore, over-expression of *TLR8* could impact its ability to carry out important roles in the host immune response. Therefore, future research should investigate the relationship between this lncRNA / mRNA to determine the impact on the host’s immune response.

The lncRNA *ENSBTAT00000084307* is predicted to interact with the upstream gene serum / glucocorticoid regulated kinase 1 (*SGK1*) which has roles in promoting glucose metabolism^56^. The role of *SGK1* in bovine mastitis is not currently known; however, previous research conducting gene expression and GWAS have shown that it is commonly DE between mastitic and healthy tissue conditions^57^. In our study, the lncRNA that is predicted to interact with *SGK1* is over-expressed in the mastitic samples (FC = 45.79, FDR = 2.16E-03) and thus, further research is warranted to better understand the relationship of this lncRNA/ mRNA in relation to mastitis or immune response. The correlation between the lncRNA and the isoform of *SGK1* (*SGKI_2*) expression levels was positive (*r* = 0.82) and significant (*P*-value = 0.05).

Lastly, *ENSBTAT00000070418_2* and *ENSBTAT00000082333* and their predicted targets were both previously discussed as they were the most over-expressed and under-expressed annotated lncRNA and therefore, not be further mentioned. Additionally, 5 of the lncRNAs in Table 2 have the same predicted targets of other DE lncRNA previously discussed throughout the manuscript, therefore, their functions will not be discussed. The first lncRNA, *ENSBTAT00000068605* is overlapping and predicted to interact with *TRIB1* (FC = 19.82, FDR = 2.37E-02). The second lncRNA *ENSBTAT00000069074* is predicted to interact with both *bta-mir-10163* and *EMB*, which are both located upstream from the lncRNA (FC = 25.06, FDR = 4.09E-03). Only the interaction between the lncRNA and EMB was significantly positively correlated (*r* = 0.94, *P*-value = 0.00) in the healthy group. Lastly, the lncRNAs *ENSBTAT00000070418_1, ENSBTAT00000070418_3* and *ENSBTAT00000070418_4* were predicted to interact with the downstream gene *PIK3R4* and are all over-expressed in the mastitic samples compared to the healthy samples (FC = 25.41, FDR = 3.06E-03; FC = 46.89, FDR = 4.37E-02; FC = 27.38, FDR = 3.14E-03, respectively). When looking at the correlations, there were significant interactions between *ENSBTAT00000070418_1* and *PIK3R4_1* (*r* = 0.83; *P*-value = 0.04) in the healthy group*, ENSBTAT00000070418_1* and *PIK3R4_3* (*r* = 0.94; *P*-value = 0.01) in the mastitic group and *ENSBTAT00000070418_2* in the healthy group (*r* = −0.82; *P*-value = 0.05; Supplementary Table 2).

### Functional analysis of novel differentially expressed lncRNA

As mentioned earlier, novel lncRNA were split into intergenic and genic. Therefore, functional analysis was completed separately for both groups using the platform NetworkAnalyst. It was hypothesized that some of the lincRNAs and genic lncRNAs act in close proximity to mRNAs involved in immune system pathways. As such, if the lncRNA is acting to suppress or enhance the mRNA, this could negatively impact the cow’s immune response and increase susceptibility to mastitis infection, preventing her from being able to efficiently eliminate the threat.

### Functional analysis of novel differentially expressed long intergenic non-coding RNA

**Fig. 1:**
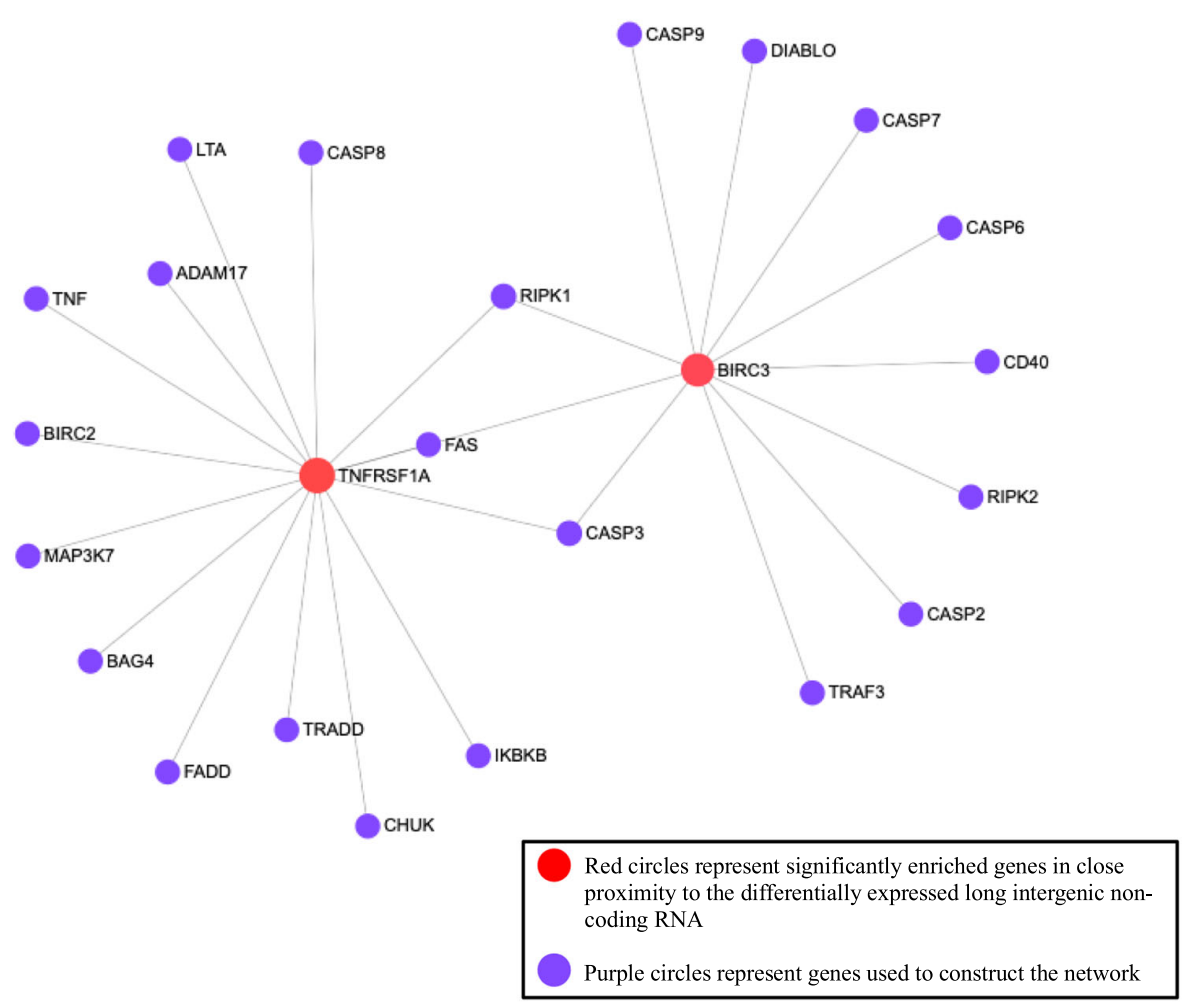
Gene network analysis constructed with the predicted interactions of the 56 long intergenic non-coding RNA (lincRNAs) using NetworkAnalyst. The two red circles represent two significantly enriched genes, which explain the majority of the topology of the network. Both tumor necrosis factor receptor superfamily member 1A *(TNFRSF1A)* and baculoviral IAP repeat containing 3 (*BIRC3)* are located in close proximity to two of the identified DE lincRNA. The purple circles represent genes used to construct the network.

Using the list of transcript interactions associated with the DE lincRNAs (N = 56; Table 3) to perform the functional analysis, 44 significantly enriched metabolic pathways were identified (FDR < 0.05; Table 4). The significant metabolic pathways were associated with immune mechanisms such as positive regulation of immune response, regulation of cytokine biosynthetic process and T-cell differentiation, among others. Two of the lincRNAs (*lincRNA_2411*.6 and *lincRNA_1450.2*) act in close proximity to two genes, tumor necrosis factor receptor superfamily member 1 A (*TNFRSF1A*) and baculoviral IAP repeat containing 3 (*BIRC3*), which explain the majority of the topology of the network (Fig. 1).

**Table 4:** Significantly enriched pathways for genes associated with differentially expressed long intergenic non-coding RNA (lincRNA).

Metabolic pathway	Total (*N*)^a^	Total genes associated with lincRNA (*N*)^b^	*P*-value
Steroid biosynthetic process	215	11	3.87E-14
Positive regulation of immune response	130	9	9.27E-13
Intracellular transport	37	5	6.52E-09
Apoptotic nuclear changes	178	7	2.57E-08
Isoprenoid metabolic process	60	5	7.95E-08
Multicellular organismal development	44	4	1.29E-06
Regulation of immune response	90	4	2.30E-05
Regulation of cytokine biosynthetic process	708	7	2.40E-04
Mitochondrion organization	17	2	4.69E-04
Response to hypoxia	105	3	1.01E-03
Protein tetramerization	27	2	1.20E-03
Positive regulation of transcription, DNA_dependent	28	2	1.29E-03
Intracellular signal transduction	29	2	1.38E-03
Transcription initiation from RNA polymerase II promoter	480	5	1.80E-03
Epithelial cell differentiation	34	2	1.90E-03
Regulation of cyclin_dependent protein kinase activity	52	2	4.39E-03
Superoxide metabolic process	54	2	4.72E-03
Lipid biosynthetic process	374	4	5.06E-03
Sulfur compound metabolic process	222	3	8.36E-03
Regulation of chromosome organization	5	1	9.56E-03
Vitamin metabolic process	82	2	1.06E-02
T-cell differentiation	83	2	1.09E-02
Macromolecule biosynthetic process	252	3	1.18E-02
Negative regulation of programmed cell death	7	1	1.34E-02
Regulation of transcription from RNA polymerase II promoter	498	4	1.37E-02
Chromosome condensation	9	1	1.71E-02
Protein_DNA complex assembly	10	1	1.90E-02
Regulation of JAK_STAT cascade	112	2	1.92E-02
Cellular response to extracellular stimulus	11	1	2.09E-02
DNA damage response, signal transduction by p53 class	317	3	2.17E-02
Regulation of neurotransmitter levels	12	1	2.28E-02
Positive regulation of I_kappaB kinase/NF_kappaB cascade	12	1	2.28E-02
Negative regulation of myeloid cell differentiation	124	2	2.32E-02
Myeloid cell differentiation	14	1	2.65E-02
Cellular cation homeostasis	15	1	2.84E-02
Establishment of vesicle localization	15	1	2.84E-02
Regulation of intracellular transport	153	2	3.43E-02
Sphingolipid biosynthetic process	19	1	3.59E-02
Negative regulation of catalytic activity	19	1	3.59E-02
Heme biosynthetic process	165	2	3.93E-02
Positive regulation of JUN kinase activity	21	1	3.96E-02
Regulation of action potential	22	1	4.14E-02
Regulation of actin polymerization or depolymerization	22	1	4.14E-02
Interleukin_1 secretion	24	1	4.51E-02

^a^Total genes from NetworkAnalyst database involved with significantly enriched metabolic pathway.

^b^Total genes associated with our list of differentially expressed long intergenic non-coding RNA (lincRNA) that are involved in the significantly enriched metabolic pathways.

The *lincRNA_2411*.6 was 11 × over-expressed in the mastitic samples compared to the healthy samples (FC = 11.31, FDR = 3.19E-02) and is predicted to interact with *TNFRSF1A* which is located upstream. This receptor encodes the type 1 receptor for tumor necrosis factor - α (TNFα)^58^, which is a pro-inflammatory cytokine secreted by inflammatory cells^59^. Numerous studies have investigated *TNF*α in relation to mastitis and have found that concentrations increase during mastitis caused by *E*. *coli* and its endotoxin^60^. Other studies have identified polymorphisms in TNFα that caused an amino acid sequence change and made animals more susceptible to mastitis^61^. Tumor necrosis factor receptor superfamily member 1 A *TNFRSF1A* is also a central gene connecting numerous other immune genes such as inhibitor of nuclear factor kappa B kinase subunit beta (*IKBKB*), which regulates multiple aspects of the innate and adaptive immune system^62^ and cluster of differentiation 40 (*CD40*) that is induced by proinflammatory stimuli^63^. Thus, the close connection of this lincRNA (*lincRNA_2411*.6) with *TNFRSF1A* may potentially affect the functionality of this gene and other genes connected to this central node. We observed a significantly strong positive correlation between *lincRNA_2411*.6 and the isoform *TNFRSF1A_2* (*r* = 0.92, *P*-value = 0.01; Supplementary Table 2).

The baculoviral IAP repeat containing 3 (*BIRC3*) gene also explained the majority of the topology of the network and is located upstream of the novel lincRNA *lincRNA_1450.2*. This lincRNA is 6.88 × over-expressed in the mastitic samples compared to the healthy samples (FC = 6.88, FDR = 1.60E-02). This gene is also commonly known as cellular inhibitor of apoptosis 2 (*cIAP2*). The inhibitors of apoptosis family of proteins have numerous biological functions such as cell proliferation, cell migration, apoptosis, and regulation of innate immunity and inflammation^64^. In an intramammary infection, one aspect of the host’s innate immunity are neutrophils and neutrophils act as the first line of defense to protect the mammary gland through phagocytosis and intracellular killing of bacterial pathogens. Once the mammary gland has cleared the pathogens, apoptosis of the neutrophils is critical to limit the inflammation in the udder and return it back to normal to prevent permanent scarring in the mammary gland, which results in a loss of milk production^65,66^. This central gene is also connected to numerous caspase (*CASP*) genes including *CASP3*, *CASP7,* and *CASP9*, which function mainly in programmed cell death^67^. Programmed cell death is important to ensure that pathogen - infected cells actively initiate cell death to prevent the pathogens, such as *S. aureus*, from multiplying and spreading, causing the mastitis infection to worsen^68^. Therefore, due to the central role of *BIRC3* with the immune system, further research is required to confirm if *lincRNA_1450.2* has an impact on the *BIRC3* gene functionality. However, the correlation between them was not significant.

### Functional analysis of novel differentially expressed genic long non-coding RNA

**Fig. 2:**
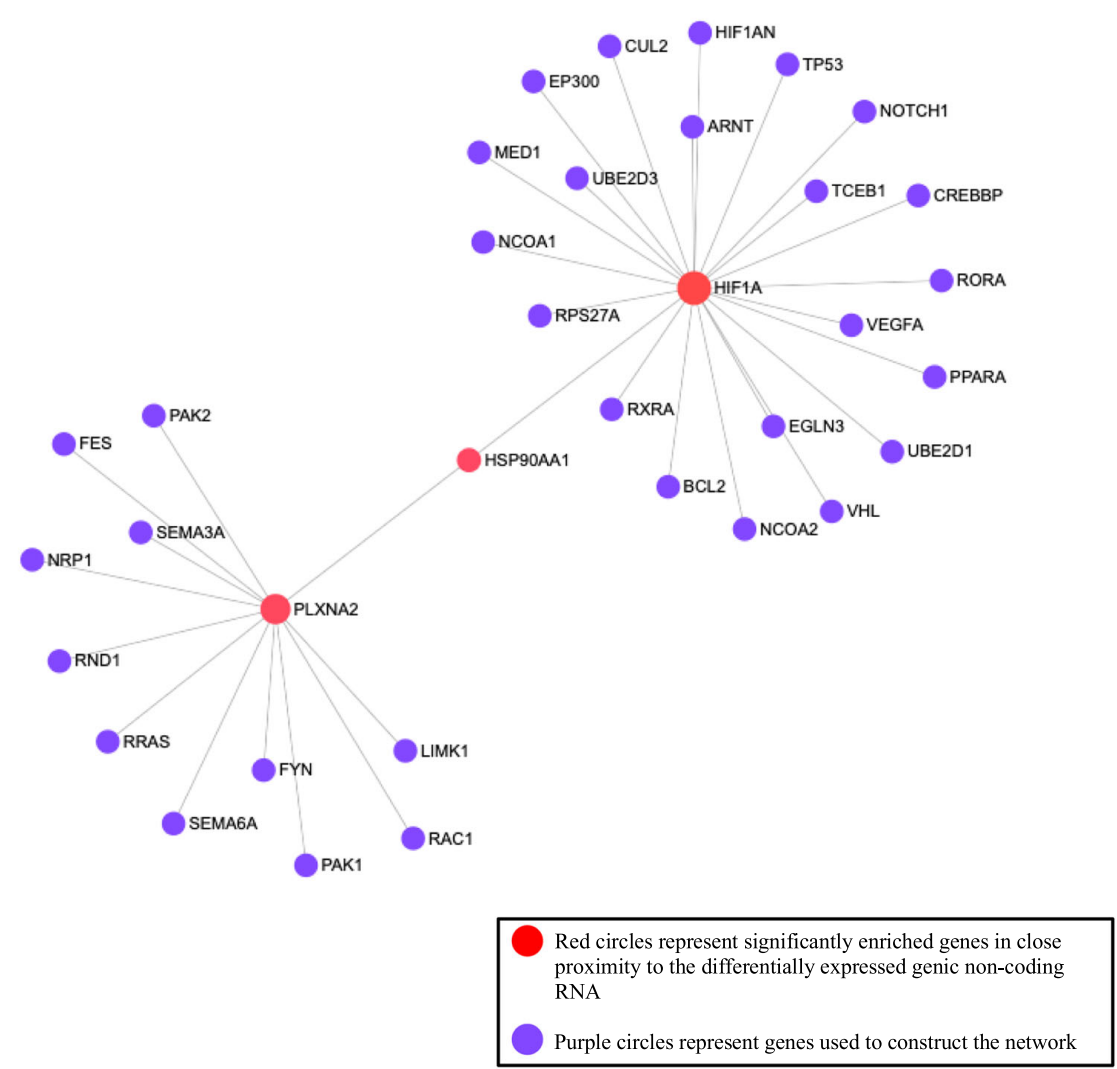
Gene network analysis constructed with the predicted interactions of the 22 genic DE lncRNAs using NetworkAnalyst. The three red circles represent three significantly enriched genes that explain the majority of the topology of the network. Both Plexin - A2 (*PLXNA2)* and hypoxia-inducible factor 1 - alpha (*HIF1A)* are located in close proximity to three of the identified DE genic lncRNA. The purple circles represent genes used to construct the network.

Using the list of mRNAs associated with 22 DE genic lncRNAs (Table 3), 65 significant metabolic pathways were identified such as inflammatory response and regulation of cytokine biosynthetic process (FDR < 0.05; Table 5). Three genes explain the majority of the topology of the network analysis, however only hypoxia-inducible factor 1 - alpha (*HIF1A* or *HIF - 1α*) and Plexin - A2 (*PLXNA2*) are in close proximity with DE genic lncRNAs (Fig. 2).

**Table 5:** Significantly enriched pathways for genes associated with the differentially expressed genic long non-coding RNA identified from the 12 milk somatic cell samples collected from 6 Holstein dairy cows.

Metabolic pathway	Total (*N*)^a^	Total genes associated with genic lncRNA (*N*)^b^	*P*-value
Cell maturation	426	9	6.73E-07
Carbohydrate transport	146	6	1.50E-06
RNA splicing, via transesterification reactions	121	5	1.22E-05
Mitotic spindle organization	88	4	6.89E-05
Positive regulation of cysteine_type endopeptidase in apoptotic process	42	3	1.61E-04
Behavior	48	3	2.40E-04
Positive regulation of T-cell proliferation	126	4	2.77E-04
Regulation of cytokine biosynthetic process	708	8	2.87E-04
Anion transport	53	3	3.22E-04
Positive regulation of cytokine secretion	1160	10	3.79E-04
Actin polymerization or depolymerization	140	4	4.14E-04
Cell morphogenesis involved in differentiation	63	3	5.37E-04
DNA replication initiation	604	7	6.44E-04
Actin filament_based process	161	4	7.01E-04
Lipid catabolic process	17	2	8.38E-04
G2/M transition of mitotic cell cycle	77	3	9.66E-04
Apoptotic nuclear changes	178	4	1.02E-03
DNA damage response, signal transduction by p53 class mediator	317	5	1.12E-03
DNA damage checkpoint	487	6	1.23E-03
Anatomical structure morphogenesis	91	3	1.57E-03
Steroid biosynthetic process	215	4	2.05E-03
Regulation of cell migration	1	1	2.56E-03
CAMP_mediated signaling	34	2	3.37E-03
Lipid homeostasis	35	2	3.56E-03
Microtubule_based process	36	2	3.77E-03
Positive regulation of protein phosphorylation	2	1	5.11E-03
Reciprocal meiotic recombination	145	3	5.85E-03
Protein processing	46	2	6.09E-03
Microtubule organizing center organization	52	2	7.73E-03
Regulation of transcription from RNA polymerase II promoter	498	5	7.88E-03
Regulation of signal transduction	53	2	8.02E-03
Endothelial cell proliferation	4	1	1.02E-02
Regulation of small GTPase mediated signal transduction	4	1	1.02E-02
Lysosomal transport	62	2	1.09E-02
Apoptotic signaling pathway	63	2	1.12E-02
Steroid metabolic process	349	4	1.14E-02
Monocarboxylic acid transport	67	2	1.26E-02
Lipid biosynthetic process	374	4	1.44E-02
Dephosphorylation	6	1	1.53E-02
Tube development	77	2	1.64E-02
Cytoskeleton organization	214	3	1.68E-02
Inflammatory response	80	2	1.76E-02
Negative regulation of DNA replication	7	1	1.78E-02
Negative regulation of phosphorylation	7	1	1.78E-02
Regulation of binding	83	2	1.89E-02
Peroxisome organization	236	3	2.18E-02
Regulation of gene expression, epigenetic	9	1	2.28E-02
Neuron development	97	2	2.53E-02
Macromolecule biosynthetic process	252	3	2.59E-02
Ras protein signal transduction	100	2	2.68E-02
Positive regulation of cell proliferation	11	1	2.78E-02
Response to hypoxia	105	2	2.93E-02
Response to hormone stimulus	12	1	3.03E-02
Neutral amino acid transport	12	1	3.03E-02
Lipoprotein metabolic process	13	1	3.28E-02
Regulation of cellular component size	14	1	3.52E-02
Protein transport	15	1	3.77E-02
B-cell differentiation	15	1	3.77E-02
Regulation of cytokine production	16	1	4.02E-02
Gamete generation	16	1	4.02E-02
Viral reproduction	17	1	4.26E-02
Metal ion transport	17	1	4.26E-02
Angiogenesis	19	1	4.75E-02
Striated muscle contraction	20	1	5.00E-02
Response to wounding	20	1	5.00E-02

^a^Total genes from NetworkAnalyst database involved with significantly enriched metabolic pathway.

^b^Total genes associated with our list of differentially expressed genic long non-coding RNA that are involved in the significantly enriched metabolic pathways.

The *HIF - 1α* gene has been detected in almost all innate and adaptive immune populations and the HIF transcription factors are key elements in immune cell metabolism and function^69^. Previous studies have shown that *HIF - 1α* are induced by pro-inflammatory cytokines such as *TNF - α* and *IL - 1β*^70^ and the expression of both increase during cases of mastitis^60,71^. As illustrated in Fig. 2 this gene is a central node for 22 other genes, some of which are immune genes (e.g., vascular endothelial growth factor A (*VEGFA*), b - cell lymphoma 2 (*BCL2*)) and some of which are involved in regulating normal cell function (e.g., mediator complex subunit 1 (*MED1*), retinoic acid receptor - related orphan receptor alpha (*RORA*)). Given the variety of functions these central genes have, it is important to consider how the DE genic lncRNAs associated with them act to enhance or impact their expression. In our analysis the *HIF - 1α* gene was associated with two DE lncRNAs (*lncRNA_577*.1 and *lncRNA_577.4*) which overlap with the exonic region of this gene. Both of these lncRNA were over-expressed in the mastitic samples in comparison to the healthy samples (FC = 6.93, FDR = 4.55E-02; and FC = 5.59, FDR = 4.47E-02, respectively). These transcripts may be a potential regulator of *HIF - 1α* gene, but further research is needed to confirm this. Additionally, in our dataset, the correlation between *HIF - 1α* and both *lncRNA_577*.1 and *lncRNA_577.4* was not significant.

Genic *lncRNA_2901.1* (FC = 7.12, FDR = 4.81E-02) was over-expressed in the mastitic samples compared to the healthy samples and is predicted to interact with *PLXNA2* as it is nested in an intronic region of the gene. This central node (Fig. 2) is connected to 11 other genes which are mainly involved in normal cell function. Plexins are a family of proteins that act as receptors for semaphorins, which are extracellular signaling proteins, essential in the development and maintenance of organs and tissues^72^. Plexins and semaphorins have also been found to mediate critical processes to the immune system including cytokine secretion, migration and cell - cell contact^73^. However, no studies have directly linked *PLXNA2* with mastitis, so further research into this specific plexin is needed and how the DE lncRNA could impact its expression.

### QTL annotation and enrichment analysis

**Fig. 3:**
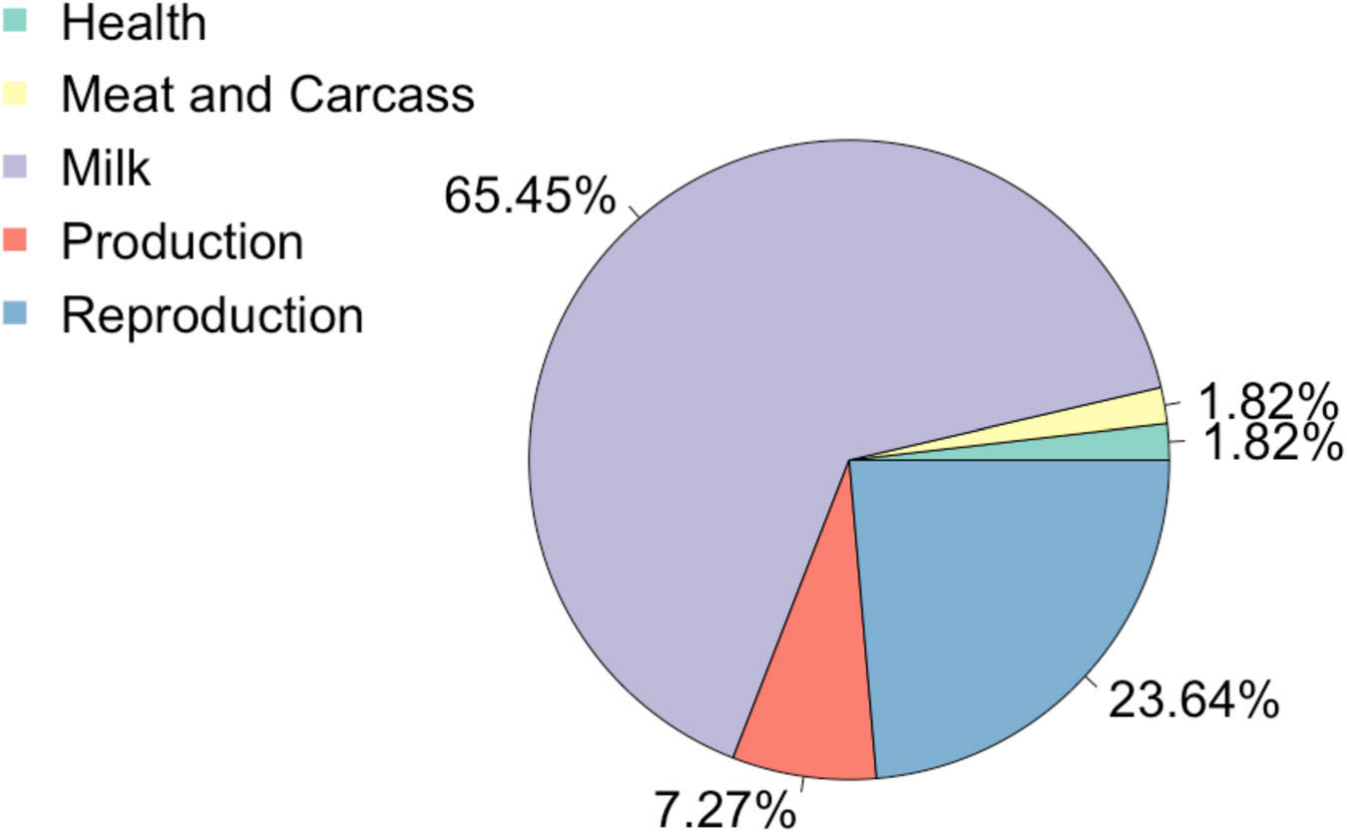
Percentage of QTL type for the QTL annotation analysis completed using the genomic regions of the 94 DE lncRNAs using GALLO R package. The pie chart represents the percentages of each QTL type from the annotation analysis. The slices represent as follows: purple = milk, blue = reproduction, red = production, green = health and yellow = meat and carcass.

The current cattle QTL database has 159,844 QTL reported, relating to 653 different traits (release 42; https://www.animalgenome.org/cgi-bin/QTLdb/index)^74^. Previous research by Tong et al.^5^ reported that several QTLs in regions of lncRNAs affect clinical mastitis, milk quality or production. In our study, 31 QTL were previously annotated within the regions of the 94 DE lncRNAs (Table 6). These QTL were associated with milk (65%), reproduction (24%), production (7%), health (2%) and meat/carcass (2%; Fig. 3). The majority of QTL within the lncRNA regions were associated with milk, milk kappa - casein percentage and milk protein percentage (Supplementary Fig. 1) which is to be expected as the majority of QTL reported in the cattle QTL database are associated with production traits. However, when the QTL associated with health trait was plotted, the only QTL annotated was for ketosis and the lincRNA annotated in this region is *lincRNA_2322.5* (3:104443741-104466628). Ketosis is another highly prevalent disease cows face in early lactation when energy expenditure is higher than the dietary intake^75,76^. Some studies have studied the effects of ketosis on mastitis and other diseases, for example Uyarlar et al. found that the incidence of mastitis, metritis and the coexistence of both infections was significantly (*p* < 0.01) higher in subclinically and clinically ketotic cows^75^. Therefore, this QTL could make the cows more susceptible to ketosis and in turn, other diseases common in the negative energy balance, including mastitis.

**Table 6:** QTL annotation analysis within genomic regions of differentially expressed long non-coding RNA identified from the 12 milk somatic cells samples collected from 6 Holstein dairy cows.

lncRNA ID	Position	FC (log10)^a^	QTL category	QTL trait	Flank Markers
*lincRNA_2322.5*	3:104,443,741–104,466,628	34.54	Health	Ketosis	rs43366814
*lincRNA_2586.2*	10:4,275,621–4,325,006	9.19	Meat and carcass	Carcass weight	rs29022216
*ENSBTAT00000082333*	6:85,666,003–85,671,517	−39.49	Milk	Milk protein %	rs137600734, rs132724570, rs110274757, rs108990141
*ENSBTAT00000082333*	6:85,666,003–85,671,517	−39.49		Milk unglycosylated kappa-caesin %	rs137600734, rs132724570, rs110274757, rs108990141
*ENSBTAT00000082333*	6:85,666,003–85,671,517	−39.49		Milk glycosylated kappa-caesin %	rs137600734, rs132724570, rs110274757, rs108990141
*ENSBTAT00000082333*	6:85,666,003–85,671,517	−39.49		Milk kappa-casein %	rs137600734, rs132724570, rs110274757, rs108990141
*ENSBTAT00000082333*	6:85,666,003–85,671,517	−39.49		Milk alpha-S1-casein %	rs476634251, rs110183394, rs209300442
*ENSBTAT00000082333*	6:85,666,003–85,671,517	−39.49		Milk kappa-casein content	rs133766898, rs137600734, rs108990141, rs110274757, rs132724570
*ENSBTAT00000082333*	6:85,666,003–85,671,517	−39.49		Milk beta-casein %	rs477726713, rs460632713, rs385605865
*ENSBTAG00000050065*	20:70,033,246–70,035,651	−35.43		Milk protein %	rs41961266
*lncRNA_577.1*	10:73,857,290–73,886,496	6.93		Milk glycosylated kappa-casein %	rs135085707
*lncRNA_2570.1*	9:72,403,793–72,417,951	7.01		Milk yield	rs41594143
*lincRNA_2174.2*	1:2,129,653–2,161,384	7.12		Milk kappa-casein %	rs135537220, rs133418963, rs137196899
*ENSBTAT00000070418*	1:151,342,531–151,427,134	16.31		Milk protein yield	rs109452554
*ENSBTAT00000069074*	20:28,801,242–28,823,133	19.02		Milk protein %	rs132878568
*ENSBTAT00000069074*	20:28,801,242–28,823,133	19.02		Milk fat %	rs132878568
*ENSBTAT00000069074*	20:28,791,624–28,829,693	25.06		Milk protein %	rs132878568
*ENSBTAT00000069074*	20:28,791,624–28,829,693	25.06		Milk fat %	rs132878568
*ENSBTAT00000070418*	1:151,346,789–151,359,836	27.38		Milk protein yield	rs109452554
*ENSBTAT00000070418*	1:151,346,789–151,359,836	46.89		Milk protein yield	rs109452554
*lincRNA_200.6*	3:119,431,372–119,434,064	8.62	Production	Average daily gain	rs135897656
*lincRNA_3182.1*	22:53,273,501–53,288,313	10.66		Body weight gain	rs42017220
*lncRNA_1751.3*	19:48,405,247–48,407,474	26.13		Body weight (yearling)	rs109044085
*lncRNA_1751.3*	19:48,405,247–48,407,474	26.13		Body weight gain	rs109044085
*lincRNA_2165.1*	X:98,097,707–98,098,234	10.29	Reproduction	Scrotal circumference	rs135056594
*lincRNA_2165.1*	X:98,097,707–98,098,234	10.29		Age at puberty	rs135056594
*lncRNA_1505.1*	16:44,572,821–44,616,632	496.05		Interval to first estrus	rs41608555
*lincRNA_2159.2*	X:79,073,608–79,080,933	683.48		Age at puberty	rs132656042, rs137169584, rs135758570
*lincRNA_2159.2*	X:79,073,608–79,080,933	683.48		Scrotal circumference	rs132656042, rs137169584
*lincRNA_2159.2*	X:79,073,608–79,080,933	683.48		Scrotal circumference	rs135758570
*lincRNA_2159.2*	X:79,073,608–79,080,933	683.48		Normal sperm %	rs132656042, rs137169584, rs135758570

^a^FC (log10) = Fold change (log10).

**Fig. 4:**
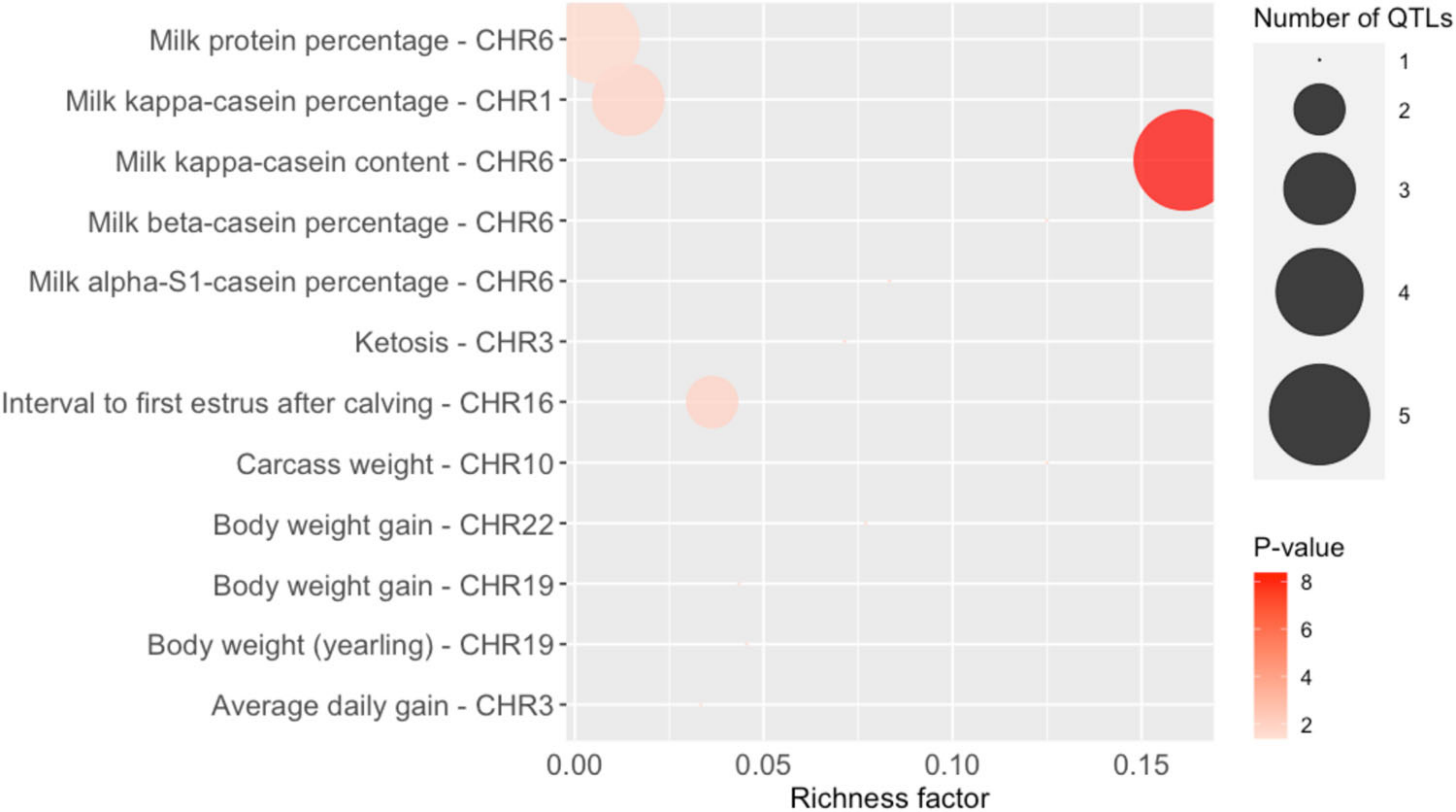
Bubble plot displaying the QTL enrichment results for the enriched QTLs identified using the genomic regions of the 94 DE lncRNAs. The area of the bubbles represents the number of observed QTL for that QTL class, and the color represents the *P*-value scale (darker color = smaller *P*-value). The richness factor for each QTL represents the ratio of the number of QTL and the expected number of QTL.

Additionally, QTL enrichment analysis was performed to correct for the large proportion of existing studies which have evaluated milk traits in dairy cattle^77,78^. As shown in the bubble plot in Fig. 4, the milk production traits were still the most enriched traits, as the number of associated studies for a specific trait can directly influence the enrichment results (Supplementary Table 3). Although QTL directly related to mastitis (e.g., somatic cell count, somatic cell score, clinical mastitis) were not found, previous research has shown genetic and phenotypic correlations between mastitis and milk production related traits. Milk yield is a trait with a genetically unfavorable correlation with clinical mastitis^79,80^. Additionally, the abundance of numerous milk proteins changes during cases of mastitis, which is unfavorable for the producer^81^. As there are numerous QTL within the lncRNAs genomic regions associated with milk production traits, careful consideration must be made before targeting specific genomic regions for breeding purposes. However, studying these regions could provide a deeper insight into the potential functionality of the lncRNAs identified in this study, in relation to both mastitis resistance and milk production traits.

## Conclusions

RNA - Sequencing analysis was used to identify DE lncRNAs between 6 healthy and 6 mastitic milk somatic cell samples. The previously annotated lncRNAs identified in this study were predicted to interact with mRNA and miRNA that are involved in immune pathways and could impact the host’s immune response to mastitis. Functional candidate novel lncRNAs were identified due to their involvement with the immune system and acted as central nodes in immune pathways, specifically those interacting with hypoxia - inducible factor 1 - alpha (*HIF - 1*α) which is detected in most innate and adaptive immune populations, plexin - A2 *(PLXNA2)* which mediates critical processes to the immune system, tumor necrosis factor receptor superfamily member 1 A *(TNFRSF1A)* which is involved with numerous other immune genes, and lastly baculoviral IAP repeat containing 3 (*BIRC3)* which helps regulate innate immunity and inflammation. The QTLs in the lncRNA genomic regions are associated with numerous milk traits, such as milk protein percentage, as well as in the QTL region associated with ketosis. Therefore, studying the transcriptome at a high - throughput level using RNA – Seq enabled the identification of lncRNA regulatory elements that have a potential functional role in immune response to mastitis in Holstein dairy cows. In turn, this research could aid in the development of breeding programs to select animals that are more resistant to mastitis infections.

## Methods

### Animal material and sample collection

This study was approved by the UC Davis Institutional Animal Care and Use Committee (IACUC). Sample collections and procedures were performed in accordance with the approved guidelines of UC Davis IACUC. As described in Asselstine et al., 6 Holstein dairy cows, ranging from first to third lactation from the University of California - Davis were used in this study and all 6 of these animals had natural cases of mastitis^82^. The natural cases of mastitis were diagnosed by the California mastitis test which detects early presence of mastitis in the milk based on the SC count^3,83^. The California mastitis test can be performed quickly cow-side and works by using a reagent that disrupts the cell membrane of somatic cells present in the milk sample. The DNA in the cells then reacts with the test reagent to form a gel, and the degree of gelling is positively associated with SCC in the sample^83^. Generally, this test cannot detect SCC below 350,000 cells/mL. Two different samples were taken from each cow, one sample from the mastitic quarter (*N* = 6) and the other sample taken diagonally away from the mastitic quarter and classified as healthy (*N* = 6) which was verified based on having a somatic cell count <100,000 cells/mL (*N* = 12)^82^. Using examination gloves, the same technician for all the samples cleaned the cow’s teats was with gauze and damped in 70% isopropanol and 50-mL of milk sample was taken from each quarter using a 3 - cm plastic cannula (Genesis Industries Inc., Elmwood, WI) to ensure no external bacteria contaminated the sample. Milk was kept on ice and immediately processed for RNA extraction. After the milk samples were collected, the cow was immediately treated to mediate the mammary infection.

After being stored on ice, the technician then separated the pellet of SC from the upper milk fat globule membrane and the pellet was washed using RNase - free Phosphate Buffered Saline (PBS) and EDTA (50 mL PBS + 50 μL EDTA) following protocol outlined by Cánovas et al.^84^. Total RNA was purified following the Trizol protocol (Invitrogen, Carlsbad, CA); and the RNA was quantified by an ND - 1000 Nanodrop Spectrometer (Thermo Scientific, Pittsburgh, PA)^85^. All samples passed the RNA integrity number (range of 8.0–9.0), indicating good RNA quality^85^. As described in Cánovas et al.^86,87^ library construction was performed using the TruSeq RNA sample preparation kit (Illumina, San Diego, CA). Sequencing was completed at the same time by a facility technician with an Illumina HiSeq 2000 analyzer that yielded 100 - bp single - read sequences.

The data discussed in this publication have been deposited in NCBI’s Gene Expression Omnibus and are accessible through Gene Expression Omnibus series accession number GSE131607 (https://www.ncbi.nlm.nih.gov/geo/ query/acc.cgi?acc = GSE131607).

### RNA - sequencing analysis

#### Read trimming and quality control

The raw sequence data was trimmed using the automatic trimmer function of CLC Genomics Workbench (CLC Bio, Aarhus, Denmark) using a quality trimming score = 0.05. After the reads were trimmed, quality control was performed using the NGS quality control tool of CLC Genomics Workbench as described by Cánovas et al.^86^. All samples passed the quality control analysis based on GC content, Phred score and over-represented sequence parameters to name a few^82,86^.

#### Sequence assembly

The Large Gap Read Mapping (LGRM) tool in CLC Genomics Workbench was used to map the reads to the bovine reference genome (ARS_UCD1.2.1; ftp.ensembl.org/pub/release-100/fasta/bos_taurus)^88^. The LGRM tool can map sequence reads that span introns without requiring prior transcript annotation^89^. Assembly was conducted with a length fraction of 0.7 and a similarity of 0.8 to exclude paralogous sequence variants and the settings were as follows: a mismatch cost = 2, deletion cost = 3, insert cost = 3, minimum contig length = 200 base pairs (bp) were allowed^88^.

Using CLC Genomics Workbench, transcript discovery was performed to identify transcripts in each group individually. Starting with the healthy group, the transcript discovery used: 1) the bovine reference genome and 2) the LGRM assembly for the healthy group. Parameters for filtering include gene merging distance = 50, minimum reads in gene = 10 and minimum predicted gene length ≥ 200 bp^88^. For the mastitic transcript discovery, the predicted RNA and gene tracks generated from the healthy group as well as the annotated bovine reference genome were used. Thus, the predicted RNA file (.gtf) contains predicted information from both groups of samples (healthy and mastitis), in addition to the annotated genome information.

#### Long non-coding RNA identification

To identify lncRNAs, three different FIExible Extraction of LncRNAs (FEELnc) pipelines were used (FEELnc filter, FEELnc codpot and FEELnc classifier; https://github.com/tderrien/FEELnc)^90^. The FEELnc filter pipeline was used to filter and remove protein-coding, pseudogene, miRNA etc and capture transcripts with a minimal size of 200 bp. The FEELnc codpot pipeline was applied to compute the coding potential score (CPS; [0–1]) for each of the candidate transcripts in the predicted RNA file (.gtf) generated in the previous step. From this, the mRNA vs lncRNA can be separated based on their maximized specificity (Sp) and sensitivity (Sn). The CPS calculation was based on three parameters: the k - mer frequencies, which were left as default with values of: 1, 2, 3, 6, 9 and 12 mers, the Open Reading Frame (ORF) coverage and the mRNA size^91^. The CPS cut - off for the samples was 0.92 of both sensitivity and specificity (Supplementary Fig. 2). Lastly, FEELnc classifier pipeline was used to identify the best partner interaction of each lncRNA. This is determined based on different aspects including: the lncRNAs type, subtype and location. To classify the lncRNA type, lncRNA can either be ‘genic’ where they overlap with a gene, or they can be classified as ‘intergenic’ when they are located between genes (either upstream or downstream) and these are commonly referred to as (long intergenic non-coding RNA; lincRNA). For the subtype category, there are different classifications; genic lncRNA can be classified as: 1) ‘nested’ in which the lncRNA is contained in the RNA partner transcript, or (2) ‘overlapping’ meaning the lncRNA partly overlaps with the RNA partner transcript. For lincRNA, they can be classified as (1) ‘same_strand’ in which the lncRNA is transcribed in the same orientation with its RNA partner, (2) ‘divergent’ in which the lncRNA is transcribed in head-to-head orientation with the RNA partner, or (3) ‘convergent’ where the lncRNA is orientated in tail to tail with its RNA partner. Next, the location can be defined according to the orientation of the interactions and the localization of the interactions (genic can be overlapping an intronic or exonic region of the partner RNA; lincRNA can be located upstream or downstream). Next, to determine the best partner RNA, for genic lncRNA, the best RNA partner is by rule of priority exonic, then intronic, then containing. Whereas for lincRNA, the best RNA partner is the closest to the lincRNA. For both genic and lincRNA, the best partner interaction is assigned a numerical value of 1 = best identified match for the lncRNA or 0 = indicating there is a better match. Once this is determined, functional annotation and relationships between lncRNA and their predicted target interactions, e.g., mRNA or miRNA is identified^90^.

From the previous step, a file is generated containing the identified lncRNA, which was then combined with the annotated reference genome (.gtf file). Using this combined file, the trimmed reads were then aligned to the bovine reference genome using CLC Genomics Workbench (CLC Bio, Aarhus, Denmark). RNA - Sequencing analysis was then performed in CLC Genomics Workbench using mapping criteria as mentioned prior (mismatch = 2, insertion = 3, deletion costs = 3). We also used the same criteria for the length and similarity fractions (0.7 and 0.8, respectively). Expression values for the lncRNA were on a count - based model which were then transformed and normalized.

### Differential lncRNA expression analysis and classification

Differential expression analysis on the lncRNAs was performed between healthy (N = 6) and mastitic (N = 6) samples by Empirical analysis of differential gene expression tool (CLC Genomics Workbench). Transcripts were classified as DE between healthy and mastitic samples when FDR < 0.05 and a fold change (|FC|) > 2. Among the DE transcripts, only those annotated as lncRNA using the FEELnc software were used for further analysis. We were interested in looking not only at the lncRNA as a whole, but also looking at if they were 1) previously annotated for both gene name and length in the ARS_UCD1.2.1 bovine reference genome, 2) previously annotated for gene name in the ARS_UCD1.2.1 bovine reference genome and 3) novel lncRNAs for which no gene name or gene length was previously annotated in the ARS_UCD1.2.1 bovine reference genome.

### Functional analysis

Functional analysis including metabolic pathway analysis and gene networks analysis was performed on the partner interactions of the DE lncRNAs identified in this study. To determine the functions of the DE lncRNAs, the lncRNAs were split into 1) previously annotated, including those annotated for both gene name and length and those annotated for gene name, but length was different than that reported in the bovine reference genome (ARS.UCD 1.2.1) and 2) novel lncRNAs with neither gene name nor length reported in the bovine reference genome (ARS.UCD 1.2.1).

For the previously annotated DE lncRNAs, the genomic position (e.g., chr: start - end region) of each DE lncRNA was used to identify proximal RNA transcripts through Genome Data Viewer (https://www.ncbi.nlm.nih.gov/genome/gdv/?org=bos-taurus). These proximal RNA transcripts were then considered to be the predicted target of the DE lncRNA and functional analysis was performed on a lncRNA – by – lncRNA basis.

For the novel lncRNA, as previously mentioned the FEELnc classifier pipeline was used to determine the predicted target interaction. After this predicted target was identified, the NetworkAnalyst platform was used to perform the gene network analysis (http://www.networkanalyst.ca), using the list of predicted target interactions associated with the DE lncRNAs identified as the input. This software performs meta - analysis on gene expression data sets, to determine important features, patterns, functions and connections between genes^92–96^.

Significant (P-value < 0.05) correlations between the lncRNA and its predicted interaction within all categories (previously annotated (genes + length), previously annotated (genes), novel (genic) and novel (intergenic)) are shown in Supplementary Table 2. Within each category of lncRNA, a Pearson Correlation analysis was performed in SAS (SAS version 9.4) for each lncRNA and its predicted interaction to provide further insight on the relationship between their expression level patterns. The RPKM values of the predicted interactions was downloaded from the raw expression analysis in CLC genomics workbench. Each predicted interaction could have had multiple mRNA isoforms present, so the correlation analysis was performed for every potential mRNA isoform of the predicted interaction. Only statistically significant (*P-*value < 0.05) interactions were discussed in the manuscript where relevant.

### QTL annotation and enrichment analysis

Lastly, QTL annotation was performed using the R package: Genomic functional Annotation in Livestock for positional candidate LOci (GALLO)^77^. The genome coordinates of the DE lncRNAs was used, as well as the QTL gff annotation file retrieved from the cattle QTL Database (https://www.animalgenome.org/cgi-bin/QTLdb/index)^74^. Intervals of 1000 bp was used to account for 1000 bp upstream and 1000 bp downstream of each DE lncRNA coordinates^97^. Additionally, to evaluate if the QTL classes and traits identified around the selected DE lncRNA were significantly overrepresented, QTL enrichment qtl_enrich() function from GALLO was performed using the output obtained from the QTL annotation step^78,98^.

### Statistics and reproducibility

No statistical method was used to determine the minimum sample size in this study. There was no data excluded from the analysis. The experiment was not randomized and the investigators were not blinded during the experiment and outcome assessment. For comparisons between two groups (healthy and mastitic) differential expression analysis on the lncRNAs was performed using the Empirical analysis of differential gene expression tool of CLC Genomics Workbench. The program FEELnc was used to identify lncRNA from the dataset. The R package GALLO was used to perform the QTL annotation and enrichment analysis.

### Reporting summary

Further information on research design is available in the Nature Portfolio Reporting Summary linked to this article.

### Supplementary information

Supplementary Tables and Figures

Reporting Summary

## Data Availability

The datasets generated and/or analyzed during the current study are available in the NCBI’s Gene Expression Omnibus repository, https://www.ncbi.nlm.nih.gov/geo/query/acc.cgi?acc. Accession ID = GSE131607.
