# Supplementary material for: Novel lncRNA regulatory elements in milk somatic cells of Holstein dairy cows associated with mastitis
Source: Commun Biol. 2024 Jan 15;7:98. doi: 10.1038/s42003-024-05764-y (PMC10789785; doi:10.1038/s42003-024-05764-y)
Supplement: Supplementary file 1 — Supplementary Tables and Figures [file 42003_2024_5764_MOESM1_ESM.pdf]

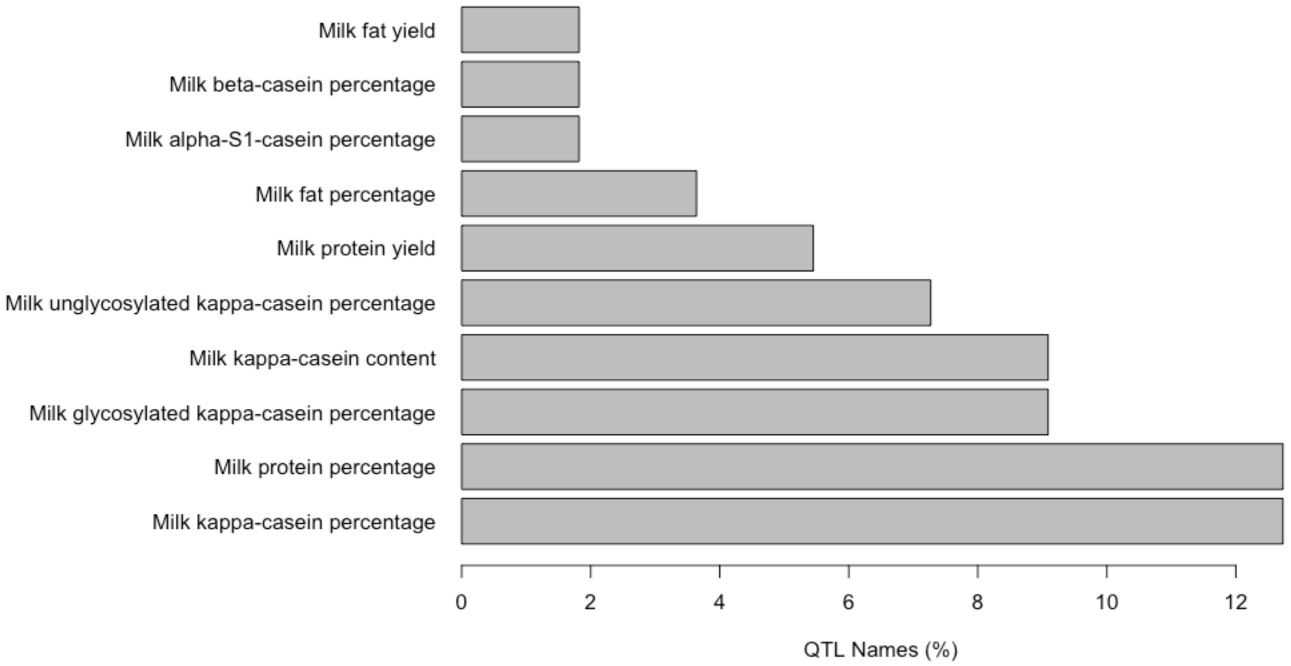

Supplementary Figure 1: The QTL in our analysis associated with the milk trait, with the most QTL being associated with milk kappa-casein percentage and milk protein percentage.

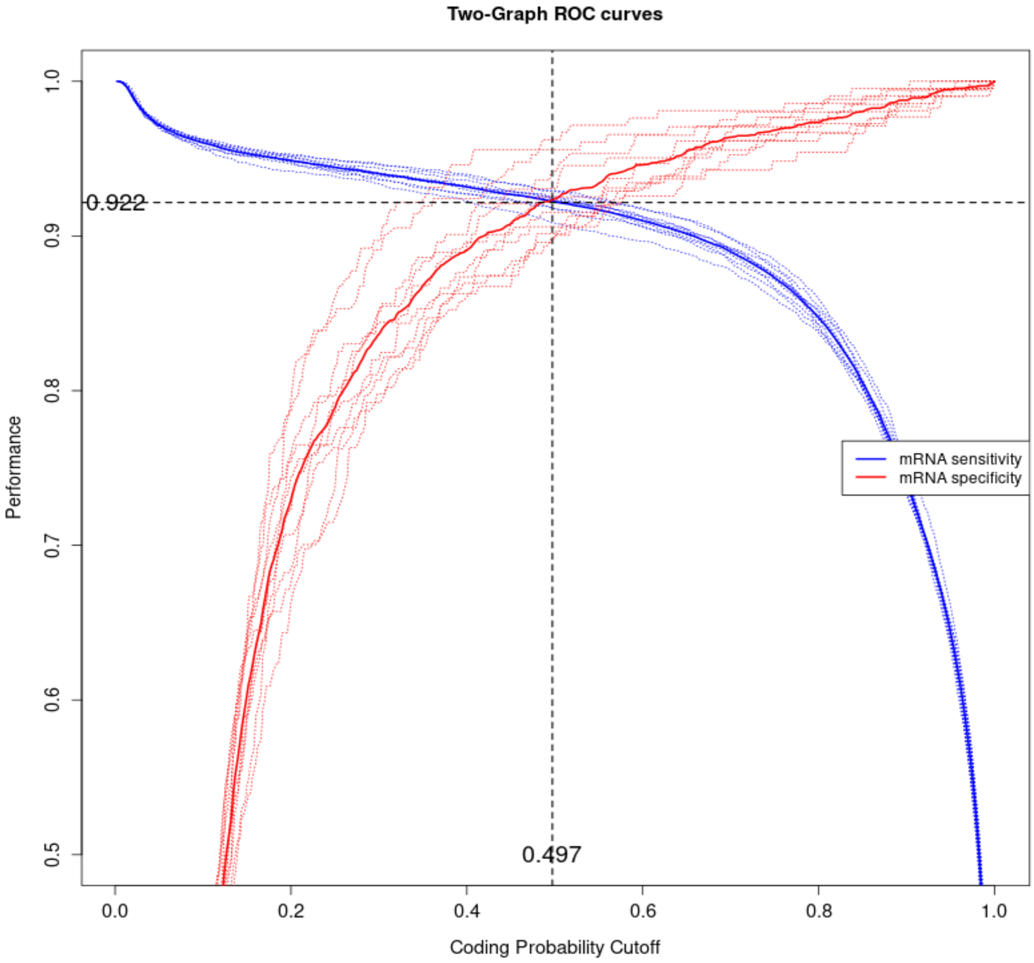

Supplementary Figure 2: The coding potential score computed using FEELnc codpot.

Supplementary Table 1: Alignment to the ARS_UCD1.2.1 bovine reference genome for the 12 milk somatic cells samples collected from 6 Holstein dairy cows

Group	Sample ID	Total reads mapped	Uniquely mapped %	Non-specifically mapped %	Unmapped %
Healthy	50A	22,791,224	91.35	5.19	3.46
	50C	12,757,084	81.84	7.5	10.67
	50E	11,257,761	85.75	5.56	8.7
	50G	9,041,681	78.7	4.87	16.43
	70A	11,737,755	87.18	12.34	0.47
	70E	16,124,047	95.17	4.36	0.48
	Average group	13,951,592 ± 2,004,582	86.66	6.63	6.7
Mastitic	50B	18,884,191	93.19	5.2	1.61
	50D	26,510,003	92.7	6.47	0.83
	50F	14,613,683	91.21	5.67	3.11
	50H	15,174,409	90.4	6.86	2.75
	70C	10,692,707	95.33	3.93	0.74
	70G	12,365,761	95.63	3.95	0.41
	Average group	16,373,459 ± 2,323,219	93.08	5.35	1.58

[1]These samples were collected from the same cow (3rd lactation, 74 DIM); [2]These samples were collected from the same cow (2nd lactation, 44 DIM); [3]These samples were collected from the same cow (3rd lactation, 178 DIM); [4]These samples were collected from the same cow (2nd lactation, 133 DIM); [5]These samples were collected from the same cow (2nd lactation, 7 DIM); [6]These samples were collected from the same cow (1st lactation, 236 DIM).

Supplementary Table 2: Significant (P-value < 0.05) correlations between the lncRNA and its predicted interaction

Category	lncRNA	Predicted Interactions	Genetic Correlations (r[1] and *P*-value)	
			Healthy	Mastitic
Previously Annotated (Gene + Length)	*ENSBTAG00000050065_2*	*IRX2_1*		0.93
				0.01
		IRX2_2	0.98	0.99
			0	0
	ENSBTAG00000051221_3	*TRIB1*	0.9	
			0.01	
Previously Annotated (Gene)	*ENSBTAT00000069074*	*EMB*	0.94	
			0	
	ENSBTAT00000070418_1	*PIK3R4_1*	0.83	
			0.04	
		PIK3R4_3		0.94
				0.01
	ENSBTAT00000070418_2	*PIK3R4_3*	-0.82	
			0.05	
	ENSBTAT00000084307	*SGK1_2*	0.82	
			0.05	
Novel (Genic)	*lncRNA_2142.1*	*ENSBTAG00000039890*	0.93	0.89
			0.01	0.02
	lncRNA_2526.1	*CTSB*	0.83	
			0.04	
	lncRNA_2570.1	*SGK1_2*		0.8
				0.05
		SGK1_3	0.82	
			0.05	
		SGK1_4	0.96	0.92
			0	0.01
	lncRNA_1930.1	*MBP_2*	0.92	
			0.01	
	lncRNA_445.5	*PDCD1LG2_1*		-0.84
				0.04
		PDCD1LG2_3	0.87	
			0.02	
		PDCD1LG2_4		-0.81
				0.05
		PDCD1LG2_5		0.79
				0.06
	lncRNA_3260.1	*LITAF*	0.9	
			0.01	
	lncRNA_2518.1	*ATP10B_3*		0.93
				0.01

	lncRNA_3370.1	*CPT1A_1*	0.87	
			0.03	
		CPT1A_3		0.99
				0
	lncRNA_1751.3	*TEX2*	0.94	0.92
			0.01	0.01
	lincRNA_64.1	*COBLL1_3*	-0.86	
			0.03	
	lincRNA_462.2	*LPL*		0.85
				0.03
	lincRNA_1670.4	*ENSBTAG00000045854_1*	0.92	0.93
			0.01	0.01
		ENSBTAG00000045854_2	0.81	0.95
			0.05	0
		ENSBTAG00000045854_3	0.92	0.94
			0.01	0.01
		ENSBTAG00000045854_5		0.82
				0.05
		ENSBTAG00000045854_6	0.86	0.84
			0.03	0.04
	lincRNA_3134.2	*BCL2A1*	0.84	
			0.04	
	lincRNA_1664.4	*ENSBTAG00000014953*	0.86	
			0.03	
	lincRNA_1668.4	*ENSBTAG00000046383*	0.83	
			0.04	
	lincRNA_2174.2	*IFNGR2_2*	0.77	
			0.07	
	lincRNA_2868.1	*PTPRJ_2*	0.93	
			0.01	
		PTPRJ_3	0.92	
			0.01	
	lincRNA_2290.2	*ENSBTAG00000054946*	0.8	0.89
			0.06	0.02
	lincRNA_495.7	*ENSBTAG00000048577_2*		0.94
				0.01
	lincRNA_2900.2	*LAMB3_2*	0.99	
			0	
**Novel (Intergenic)**	*lincRNA_3204.1*	*TREM1_1*	0.93	
			0.01	
		TREM1_2		0.82
				0.05
	lincRNA_2165.1	*bta-mir-222_1*		0.96
				0
		bta-mir-222_3	0.89	

		0.02	
lincRNA_2828.2	*ENSBTAG00000051221*		0.8
			0.06
lincRNA_3182.1	*ENSBTAG00000054150*	0.94	0.94
		0	0.01
lincRNA_2411.6	*TNFRSF1A_2*	0.92	
		0.01	
lincRNA_2517.4	*bta-mir-146a*	0.91	0.97
		0.01	0
lincRNA_2580.2	*SOD2*	0.97	
		0	
lincRNA_2289.2	*ENSBTAG00000054946*		0.83
			0.04
lincRNA_2788.1	*ENSBTAG00000049546*		0.8
			0.05
lincRNA_2517.2	*bta-mir-146a*	0.91	0.97
		0.01	0
lincRNA_3224.2	*IER3*	0.73	
		0.1	
lincRNA_2310.2	*HOOK1_1*	0.92	
		0.01	
lincRNA_2780.2	*CUL4A_1*		0.95
			0
	CUL4A_2	0.87	
		0.02	
lincRNA_343.3	*SH3BP2_2*		0.91
			0.01
	SH3BP2_5		0.82
			0.05
lincRNA_2140.1	*ENSBTAG00000006252*	0.87	
		0.02	

[1]r=correlation

Supplementary Table 3: QTL enrichment analysis within codification genomic regions of differentially expressed long non-coding RNA identified from the 12 milk somatic cells samples collected from 6 Holstein dairy cows

QTL	CHR	N_QTLs	N_QTLs_db	Total_annotated_QTLs	Total_QTLs_db	pvalue	adj.pvalue	QTL_type
Milk kappa-casein percentage	1	3	212	4	2632	1.94E-03	1.49E-02	Milk
Milk kappa-casein percentage	6	4	2490	23	20394	3.07E-01	3.36E-01	Milk
Milk protein yield	1	1	59	4	2632	8.67E-02	1.31E-01	Milk
Carcass weight	10	1	8	2	2280	7.01E-03	3.05E-02	Meat and Carcass
Milk glycosylated kappa-casein percentage	10	1	120	2	2280	1.03E-01	1.39E-01	Milk
Milk glycosylated kappa-casein percentage	6	4	1514	23	20394	8.65E-02	1.31E-01	Milk
Interval to first estrus after calving	16	2	55	2	1549	1.24E-03	1.42E-02	Reproduction
Body weight (yearling)	19	1	22	2	2102	2.08E-02	4.17E-02	Production
Body weight gain	19	1	23	2	2102	2.18E-02	4.17E-02	Production
Body weight gain	22	1	13	1	1098	1.18E-02	3.40E-02	Production
Milk fat percentage	20	1	501	3	4379	3.06E-01	3.36E-01	Milk
Milk protein percentage	20	2	1103	3	4379	1.58E-01	2.02E-01	Milk
Milk protein percentage	6	4	719	23	20394	7.95E-03	3.05E-02	Milk
Average daily gain	3	1	30	2	3011	1.98E-02	4.17E-02	Production
Ketosis	3	1	14	2	3011	9.28E-03	3.05E-02	Health
Milk alpha-S1-casein percentage	6	1	12	23	20394	1.35E-02	3.44E-02	Milk
Milk beta-casein percentage	6	1	8	23	20394	8.99E-03	3.05E-02	Milk
Milk kappa-casein content	6	5	31	23	20394	1.91E-10	4.39E-09	Milk
Milk unglycosylated kappa-casein percentage	6	4	1542	23	20394	9.12E-02	1.31E-01	Milk
Milk fat yield	9	1	48	1	1472	3.26E-02	5.77E-02	Milk
Age at puberty	X	4	9739	11	24223	7.09E-01	7.09E-01	Reproduction
Percentage normal sperm	X	3	3341	11	24223	1.85E-01	2.24E-01	Reproduction
Scrotal circumference	X	4	9393	11	24223	6.74E-01	7.05E-01	Reproduction
