# Supplementary material for: Novel lncRNA regulatory elements in milk somatic cells of Holstein dairy cows associated with mastitis
Source: Commun Biol. 2024 Jan 15;7:98. doi: 10.1038/s42003-024-05764-y (PMC10789785; doi:10.1038/s42003-024-05764-y)
Supplement: Supplementary file 2 — Reporting Summary [file 42003_2024_5764_MOESM2_ESM.pdf]

nature portfolio

Corresponding author(s): Angela Cánovas

Last updated by author(s): Dec 18, 2023

Reporting Summary

Nature Portfolio wishes to improve the reproducibility of the work that we publish. This form provides structure for consistency and transparency in reporting. For further information on Nature Portfolio policies, see our Editorial Policies and the Editorial Policy Checklist.

Statistics

For all statistical analyses, confirm that the following items are present in the figure legend, table legend, main text, or Methods section.

n/a	Confirmed	
☐	☒	The exact sample size (*n*) for each experimental group/condition, given as a discrete number and unit of measurement
☐	☒	A statement on whether measurements were taken from distinct samples or whether the same sample was measured repeatedly
☐	☒	The statistical test(s) used AND whether they are one- or two-sided<br>*Only common tests should be described solely by name; describe more complex techniques in the Methods section.*
☒	☐	A description of all covariates tested
☐	☒	A description of any assumptions or corrections, such as tests of normality and adjustment for multiple comparisons
☐	☒	A full description of the statistical parameters including central tendency (e.g. means) or other basic estimates (e.g. regression coefficient) AND variation (e.g. standard deviation) or associated estimates of uncertainty (e.g. confidence intervals)
☐	☒	For null hypothesis testing, the test statistic (e.g. *F*, *t*, *r*) with confidence intervals, effect sizes, degrees of freedom and *P* value noted<br>*Give P values as exact values whenever suitable.*
☒	☐	For Bayesian analysis, information on the choice of priors and Markov chain Monte Carlo settings
☒	☐	For hierarchical and complex designs, identification of the appropriate level for tests and full reporting of outcomes
☒	☐	Estimates of effect sizes (e.g. Cohen's *d*, Pearson's *r*), indicating how they were calculated

Our web collection on statistics for biologists contains articles on many of the points above.

Software and code

Policy information about availability of computer code

Data collection	No software was used for data collection.
Data analysis	Data was analyzed using CLC Genomics Workbench, FIExible Extraction of LncRNAs (FEELnc) , NCBI viewer and NCBI blast, and NetworkAnalyst. URLs for each software or specific program information is included in the manuscript.

For manuscripts utilizing custom algorithms or software that are central to the research but not yet described in published literature, software must be made available to editors and reviewers. We strongly encourage code deposition in a community repository (e.g. GitHub). See the Nature Portfolio guidelines for submitting code & software for further information.

Data

Policy information about availability of data

All manuscripts must include a data availability statement. This statement should provide the following information, where applicable:

- Accession codes, unique identifiers, or web links for publicly available datasets
- A description of any restrictions on data availability
- For clinical datasets or third party data, please ensure that the statement adheres to our policy

The datasets generated and/or analyzed during the current study are available in the NCBI's Gene Expression Omnibus repository, https://www.ncbi.nlm.nih.gov/geo/query/acc.cgi?acc. Accession ID = GSE131607.

Human research participants

Policy information about studies involving human research participants and Sex and Gender in Research.

Reporting on sex and gender	N/A
Population characteristics	N/A
Recruitment	N/A
Ethics oversight	N/A

Note that full information on the approval of the study protocol must also be provided in the manuscript.

Field-specific reporting

Please select the one below that is the best fit for your research. If you are not sure, read the appropriate sections before making your selection.

☒ Life sciences ☐ Behavioural & social sciences ☐ Ecological, evolutionary & environmental sciences

For a reference copy of the document with all sections, see nature.com/documents/nr-reporting-summary-flat.pdf

Life sciences study design

All studies must disclose on these points even when the disclosure is negative.

Sample size	No sample size calculation was completed. We collected samples over a 1 year period to maximize the amount of animals that were diagnosed with a natural case of mastitis. To complete RNA-Sequencing analysis, 6 animals per group, for a total of 12 samples was sufficient to achieve our research goals.
Data exclusions	No data was excluded, all samples passed quality control for RNA integrity number, and read quality control.
Replication	We collected 2 samples from each cow, one from the mastitic quarter and one from the healthy quarter (6 Holstein dairy cows total, 2 samples per cow, for a total of 12 samples collected).
Randomization	We collected 2 samples from each animal, one from the mastitic quarter, and one from the healthy quarter, therefore this allocation was not random as we were working with a specific disease and classified the quarters as either mastitic, or healthy.
Blinding	Blinding was not relevant to our study as we were not testing a treatment on the animals used in the study, we were measuring expression changes in the transcriptome of two different groups of samples, healthy and mastitic.

Reporting for specific materials, systems and methods

We require information from authors about some types of materials, experimental systems and methods used in many studies. Here, indicate whether each material, system or method listed is relevant to your study. If you are not sure if a list item applies to your research, read the appropriate section before selecting a response.

Materials & experimental systems

n/a	Involved in the study	
☒	☐	Antibodies
☒	☐	Eukaryotic cell lines
☒	☐	Palaeontology and archaeology
☐	☒	Animals and other organisms
☒	☐	Clinical data
☒	☐	Dual use research of concern

Methods

n/a	Involved in the study	
☒	☐	ChIP-seq
☒	☐	Flow cytometry
☒	☐	MRI-based neuroimaging

Animals and other research organisms

Policy information about studies involving animals; ARRIVE guidelines recommended for reporting animal research, and Sex and Gender in Research

Laboratory animals	N/A
Wild animals	N/A
Reporting on sex	N/A
Field-collected samples	The animals were housed in a freestall dairy facility at the University of California–Davis, fed a total mixed ration, and supplied water ad libitum.
Ethics oversight	This study was approved by the UC Davis Institutional Animal Care and Use Committee (IACUC). Sample collections and procedures were performed in accordance with the approved guidelines of UC Davis IACUC.

Note that full information on the approval of the study protocol must also be provided in the manuscript.
